# IL-27 Alleviates Airway Inflammation and Airway Hyperresponsiveness in Asthmatic Mice by Targeting the CD39/ATP Axis of Dendritic Cells

**DOI:** 10.1007/s10753-023-01945-9

**Published:** 2023-12-20

**Authors:** Yifei Chen, Miaojuan Zhu, Jiahao Hu, Shaojun He, Shuhua Li, Bing Liu, Jiong Yang

**Affiliations:** 1 Department of Respiratory and Critical Care Medicine, Zhongnan Hospital of Wuhan University, Wuhan, China; 2 Department of Respiratory and Critical Care Medicine, The Central Hospital of Wuhan, Tongji Medical College, Huazhong University of Science and Technology, Wuhan, Hubei China

**Keywords:** allergic asthma, CD39, rxtracellular ATP, regulatory T cells.

## Abstract

Interleukin-27 receptor (IL-27R) is expressed in a variety of immune cells and structural cells, including dendritic cells. The mechanism of IL-27 in asthma has not been fully elucidated. This study aimed to examine whether IL-27 regulated the CD39/ATP axis of dendritic cells in asthma. Our results showed that in ovalbumin (OVA)-induced asthma mouse model, IL-27Rα^−/−^ asthmatic mice showed increased airway resistance, increased infiltration of inflammatory cells in lung tissue, proliferation of goblet cells, enhanced expression of Muc5 AC around airway epithelium, increased total number of cells and eosinophils, increased levels of total IgE, OVA-IgE, IL-4, IL-5, IL-13 and IL-17 A, and increased expression of transcription factors GATA-3 and RORγt in lung tissue. The expression of CD39 mRNA and protein in the lung tissue of IL-27Rα^−/−^ asthmatic mice decreased, and the expression of NLRP3, ASC and Caspase-1 in NLRP3 inflammasome components increased. The concentration of ATP was significantly increased compared with WT asthmatic mice. *In vitro* experiments showed that the expression of CD39 in lung dendritic cells of IL-27Rα^−/−^ asthmatic mice decreased, while the expression of NLRP3 inflammasome components NLRP3, ASC and Caspase-1 increased. These findings indicate that IL-27 directly and indirectly regulates immunoinflammatory responses in asthma by acting on dendritic cells CD39/ATP Axis.

**Supplementary Information:**

The online version contains supplementary material available at 10.1007/s10753-023-01945-9.

## INTRODUCTION

Bronchial asthma (asthma) is a chronic airway inflammatory disease involving a variety of cells and cellular components. It is characterized by airway hyperresponsiveness, mucus hypersecretion, and reversible airflow limitation. Asthma is a heterogeneous disease with a variety of clinical phenotypes and different immunological pathogenesis. Chronic airway inflammation is the core pathophysiological process of asthma. Interleukin (IL) family members (such as IL-4, IL-5, IL-13, and IL-17) are important factors that mediate airway inflammation. In recent years, with the in-depth study of asthma phenotypes and immunological mechanisms, at least dozens of new cytokines have been confirmed to be involved in the pathogenesis of asthma, but there is still a lack of a thorough understanding of the development mechanism of interleukins in asthma [1].

IL-27 is a multifunctional immune regulatory factor, which is differentially regulated in multiple sclerosis/experimental autoimmune encephalomyelitis (EAE), inflammatory bowel disease (IBD), rheumatoid arthritis (RA) and other immune diseases, and plays a pro-inflammatory or anti-inflammatory role. In recent years, reports on IL-27 involved in the regulation of airway inflammation and airway hyperresponsiveness in asthma have been increasing, but different studies have controversially discussed the role and mechanism of IL-27 in the pathogenesis of asthma [2].

Dendritic cells (DC) are professional antigen-presenting cells, which are widely distributed in the airway, interstitial lung, pleura, and bronchial lymph nodes. It was previously believed that dendritic cells were the key initiators to induce allergen-specific immune responses in asthma. Dendritic cells are important cells for the synthesis and secretion of IL-27. Lipopolysaccharide (LPS) can activate the Toll-like receptor (TLR) -4 of dendritic cells and induce the increase of IL-27 synthesis through signal molecules such as myeloid differentiation factor (MyD)88, nuclear factor (NF) -κB and interferon regulatory factor (IRF) -3. Dendritic cells also express the IL-27 receptor, and the IL-27 secreted by dendritic cells has an ‘autocrine action’ on the dendritic cells themselves. In different disease environments, IL-27 differentially regulates dendritic cell function in the form of ‘autocrine’ [3].

CD39 is expressed in vascular endothelial cells, dendritic cells, regulatory T cells (Treg), and other cell types, and its expression level is regulated by cytokines, oxidative stress, and other factors. CD39 has a protective effect on cells, tissues, or organs in inflammation, injury, and autoimmune diseases because it can hydrolyze ATP and inhibit the pro-inflammatory effect of dangerous signal ATP. Therefore, we hypothesized that IL-27 affects airway inflammation and airway hyperresponsiveness in asthmatic mice by affecting the dendritic CD39 /ATP axis [4].

## MATERIALS AND METHODS

### Animals and Animal Models

Normal C57BL/6 female mice (Wild Type, WT) were purchased from the Experimental Animal Research Center of Hubei Province and raised in the Animal Experimental Center of Wuhan University (SPF). The IL-27Rα gene knockout (IL-27Rα^−/−^) mice with C57BL/6 as the background were donated by Professor Cao Ju, Department of Clinical Laboratory, the First Affiliated Hospital of Chongqing Medical University, and raised in the Animal Experimental Center of Wuhan University (SPF).

6–8-week-old mice with an average body weight of about 20 g (18–22 g) were selected and sensitized twice by intraperitoneal injection with 200 μL of OVA (Sigma-Aldrich Chemical Co, St. Louis, MO, USA) sensitizing solution containing Alum adjuvant, with an interval of 2 weeks. The number of days of the first sensitization was recorded as day 0, and the number of days of the second sensitization was recorded as day 14; on days 25, 26, and 27, nasal administration was given at a fixed time for three consecutive days to stimulate. Before nasal drip, 1% pentobarbital sodium was injected intraperitoneally (60 mg/kg body weight of mice), and the stimulation was performed after the anesthesia was satisfactory. Mice in the control group were intraperitoneally injected with 200 μl PBS buffer twice on days 0 and 14. On days 25, 26, and 27, when the mice were challenged for three consecutive days, the dosage and administration methods were the same as those in the asthma group.

### Statistical Analysis

The measurement data were expressed as mean ± standard error (x (-) ± SEM). SPSS 17.0 statistical software was used to analyze the data, and Graph Pad Prism 5 software was used for drawing. When analyzing the data, the normal distribution test (Kolmogorov–Smirnov test) and the homogeneity of variance test (Levene’s Test for Homogeneity of Variance test) were first performed in groups. One-way ANOVA was used to compare the means between multiple groups. Further comparison between the two groups, the LSD test was used when the variance was homogeneous, and Dunnett’s T3 test was used when the variance was not homogeneous. When *P* < 0.05, the difference was statistically significant.

### Additional Methodological Details

For further information on materials and methods such as specimen collection, pathological section staining, quantitative real-time PCR (qPCR) detection, Western blot detection, and enzyme-linked immunosorbent assay (ELISA), please refer to the Supplementary information 1.

## RESULTS

### IL-27Rα Gene Knockout Aggravates Airway Hyperresponsiveness and Pulmonary Inflammation

**Fig. 1:**
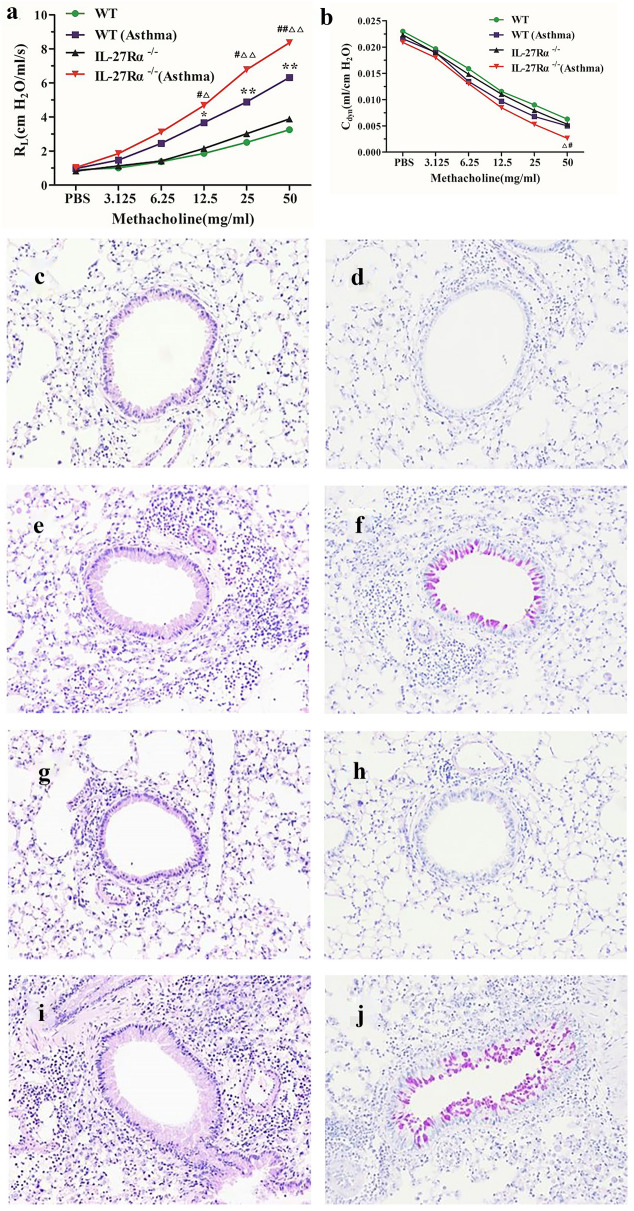
Effect of IL-27Rα gene knockout on airway hyperresponsiveness and lung inflammation in asthmatic mice. **a** The effect of twofold increasing concentration of methacholine (Mch) on airway resistance index (RI) in mice; **b** Effects of twofold increasing concentration of methacholine (Mch) on dynamic lung compliance (Cdyn) in mice. **c** and **d** HE and PAS staining of lung tissue in WT normal mice; **e** and **f** HE and PAS staining of lung tissue in WT asthmatic mice; **g** and **h** HE and PAS staining of lung tissue in IL-27Rα-/- normal mice; I and J: HE and PAS staining of lung tissue in IL-27Rα-/- asthmatic mice. * means WT group compared with WT (Asthma) group, * *P* < 0.05, * * *P* < 0.01; △ means IL-27α-/-group compared with IL-27α-/- (Asthma) group, △ *P* < 0.05, △ △ *P* < 0.01; # means WT (Asthma) group compared with IL-27α-/- (Asthma) group, # *P* < 0.05, # # *P* < 0.01.

Airway resistance index (RI) results showed that the growth rate of RI value in asthmatic mice was significantly faster than that in normal control mice. When the concentration of Mch > 12.5 mg/ml, the airway resistance of IL-27Rα^−/−^ asthmatic mice was significantly higher than that of WT asthmatic mice (*P* < 0.05 or *P* < 0.01) (Fig. 1a). The results of pulmonary dynamic compliance (Cdyn) showed that Only when the concentration of Mch reached 50 mg/ml, the Cdyn value of IL-27Rα^−^/^−^asthmatic mice was significantly lower than that of WT asthmatic mice (*P* < 0.05) (Fig. 1b).

The results hematoxylin–eosin (HE) and schiff periodic acid shiff (PAS) staining showed that the lung tissue structure of WT normal mice and IL-27Rα^−/−^normal mice was intact, and there was no obvious inflammatory cell infiltration (Fig. 1c and g). There was almost no expression of PAS-positive (PAS^+^) goblet cells in bronchial epithelial cells (Fig. 1d and h). Inflammatory cell infiltration was observed around the bronchi and small blood vessels in the lung tissue of WT asthmatic mice, mainly eosinophils, and PAS^+^ goblet cells in bronchial epithelial cells increased significantly (Fig. 1e and f). The inflammation of lung tissue in IL-27Rα^−/−^ asthmatic mice was more severe than that in WT asthmatic mice, and the PAS^+^ area normalized by a perimeter of basement membrane (PBM) was also significantly increased (Fig. 1i and j).

### IL-27Rα Gene Knockout Promotes the Expression of Muc5AC Protein in Bronchial Epithelium

**Fig. 2:**
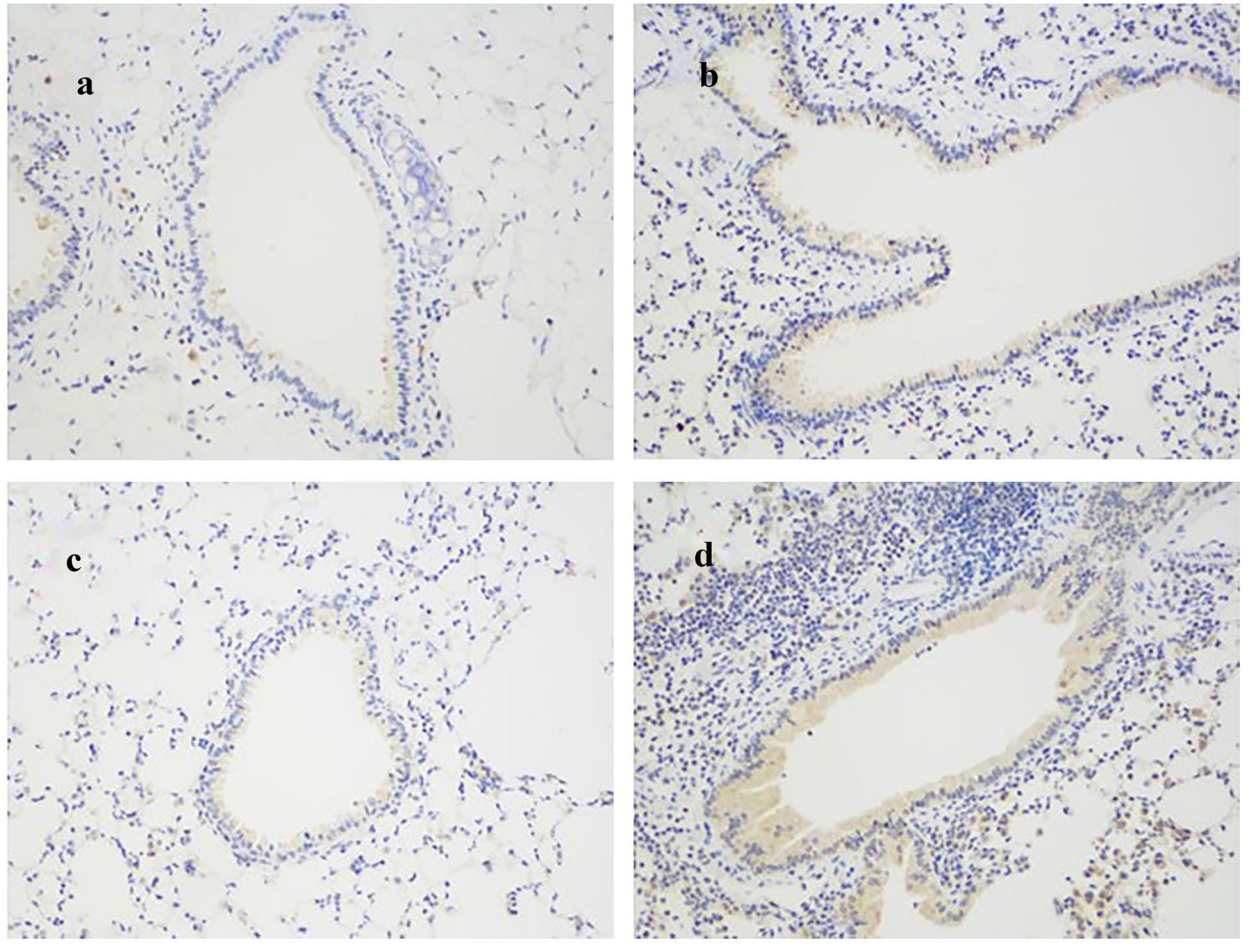
Effect of IL-27Rα gene knockout on the expression of Muc5AC protein in bronchial epithelium of asthmatic mice (× 200). **a** The expression of Muc5AC protein in bronchial epithelium of WT normal mice; **b** The expression of Muc5AC protein in bronchial epithelium of WT asthmatic mice; **c** The expression of Muc5AC protein in bronchial epithelium of IL-27Rα-/-normal mice; **d** The expression of Muc5AC protein in bronchial epithelium of IL-27Rα-/- asthmatic mice.

Semi-quantitative analysis of the average optical density value (AOD value) of Muc5AC protein in the bronchus showed that there was no significant difference in the AOD value of Muc5AC protein between WT normal mice (0.104 ± 0.008) and IL-27Rα^−/−^normal mice (0.121 ± 0.010) (*P* > 0.05). Compared with WT normal mice (0.104 ± 0.008), the AOD value of Muc5AC protein in WT asthmatic mice (0.287 ± 0.010) was significantly increased (*P* < 0.001), but the latter was still lower than that of IL-27Rα^−/−^asthmatic mice (0.345 ± 0.015), and the difference between the groups was statistically significant (*P* < 0.05). This indicates that IL-27Rα gene knockout promotes the expression of Muc5AC protein in the bronchial epithelium of mouse lung tissue (Fig. 2).

### IL-27Rα Gene Knockout Increased the Expression of Serum IgE

**Fig. 3:**
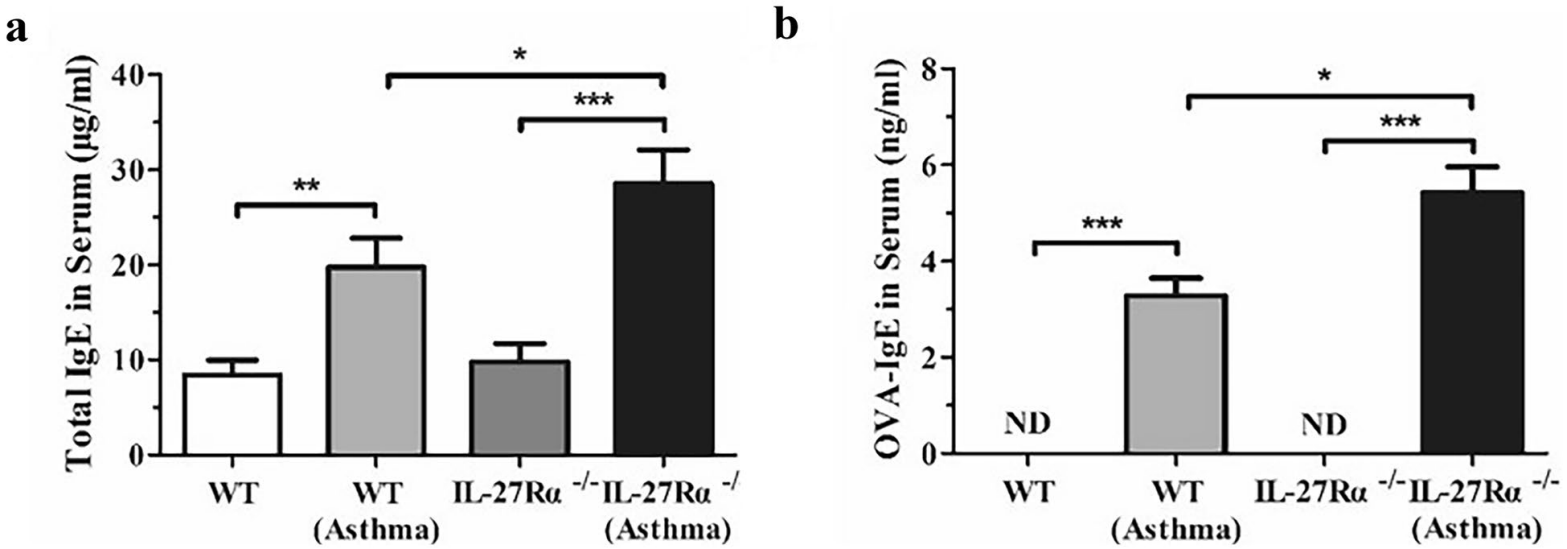
Effects of IL-27Rα gene knockout on serum total IgE and OVA-specific IgE (OVA-IgE) in asthmatic mice. **a **Serum total IgE content; **b** OVA-specific IgE content. ND (not detected): cannot be detected below the detection limit. * *P* < 0.05, * * *P* < 0.01, * * * *P* < 0.001.

The results showed that there was no significant difference in serum total IgE content between WT normal mice (8.40 ± 1.57 μg/ml) and IL-27Rα^−/−^normal mice (9.80 ± 1.92 μg/ml) (*P* > 0.05). Compared with WT normal mice (8.40 ± 1.57 μg/ml), the serum total IgE level of WT asthmatic mice (19.73 ± 3.08 μg/ml)was significantly increased (*P* < 0.01), but the latter was still lower than the serum total IgE level of IL-27Rα^−/−^asthmatic mice (28.58 ± 3.55 μg/ml), and the difference between the groups was statistically significant (*P* < 0.05) (Fig. 3a).

The serum OVA-IgE levels in WT normal mice and IL-27Rα^−/−^normal mice were lower than the lower limit of detection (< 0.32 ng/ml), while those in WT asthmatic mice and IL-27Rα^−/−^asthmatic mice were significantly increased. The serum OVA-IgE level in IL-27Rα^−/−^asthmatic mice (5.43 ± 0.53 ng/ml) was higher than that in WT asthmatic mice (3.29 ± 0.36 ng/ml), and the difference was statistically significant (*P* < 0.05) (Fig. 3b).

### Effects of IL-27Rα Gene Knockout on the BALF Cells

**Fig. 4:**
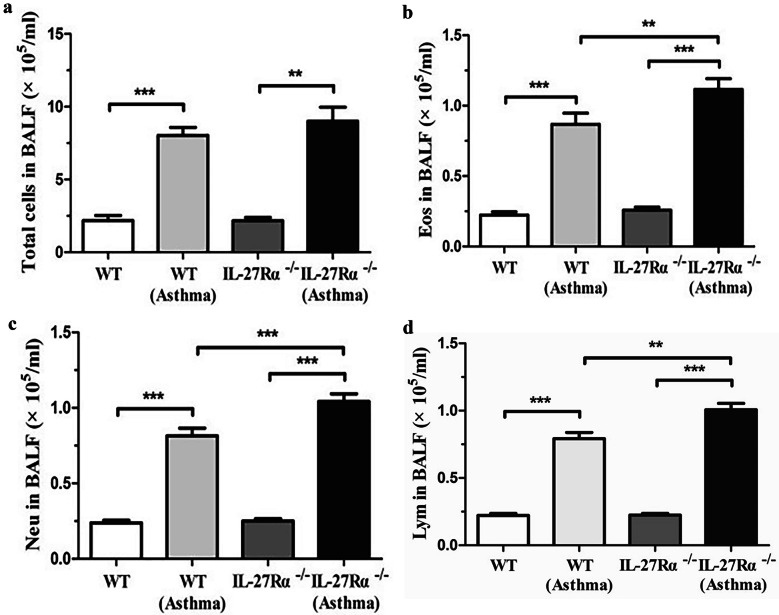
Effects of IL-27Rα gene knockout on the total number and differential count of bronchoalveolar lavage fluid (BALF) cells in asthmatic mice. **a** Total cell count in BALF; **b**, **c**, and **d** BALF cells were stained with Wright-Giemsa and counted for eosinophils (Eos), neutrophils (Neu) and lymphocytes (Lym). * * *P* < 0.01, * * * *P* < 0.001.

The results showed that there was no significant difference in the total number of BALF cells between WT normal mice and IL-27Rα^−/−^normal mic (*P* > 0.05). Compared with the above two groups, the total number of BALF cells in WT asthmatic mice and IL-27Rα^−/−^asthmatic mice increased significantly (*P* < 0.001 and *P* < 0.01), and there were significant differences in the number of different types of cells. Compared with WT asthmatic mice, the absolute values of eosinophils, neutrophils, and lymphocytes increased, and the difference was statistically significant (*P* < 0.01 or *P* < 0.001) (Fig. 4) (Supplementary information 2, Fig. 2).

### Effects of IL-27Rα Gene Knockout on Th Cell-related Factors

**Fig. 5:**
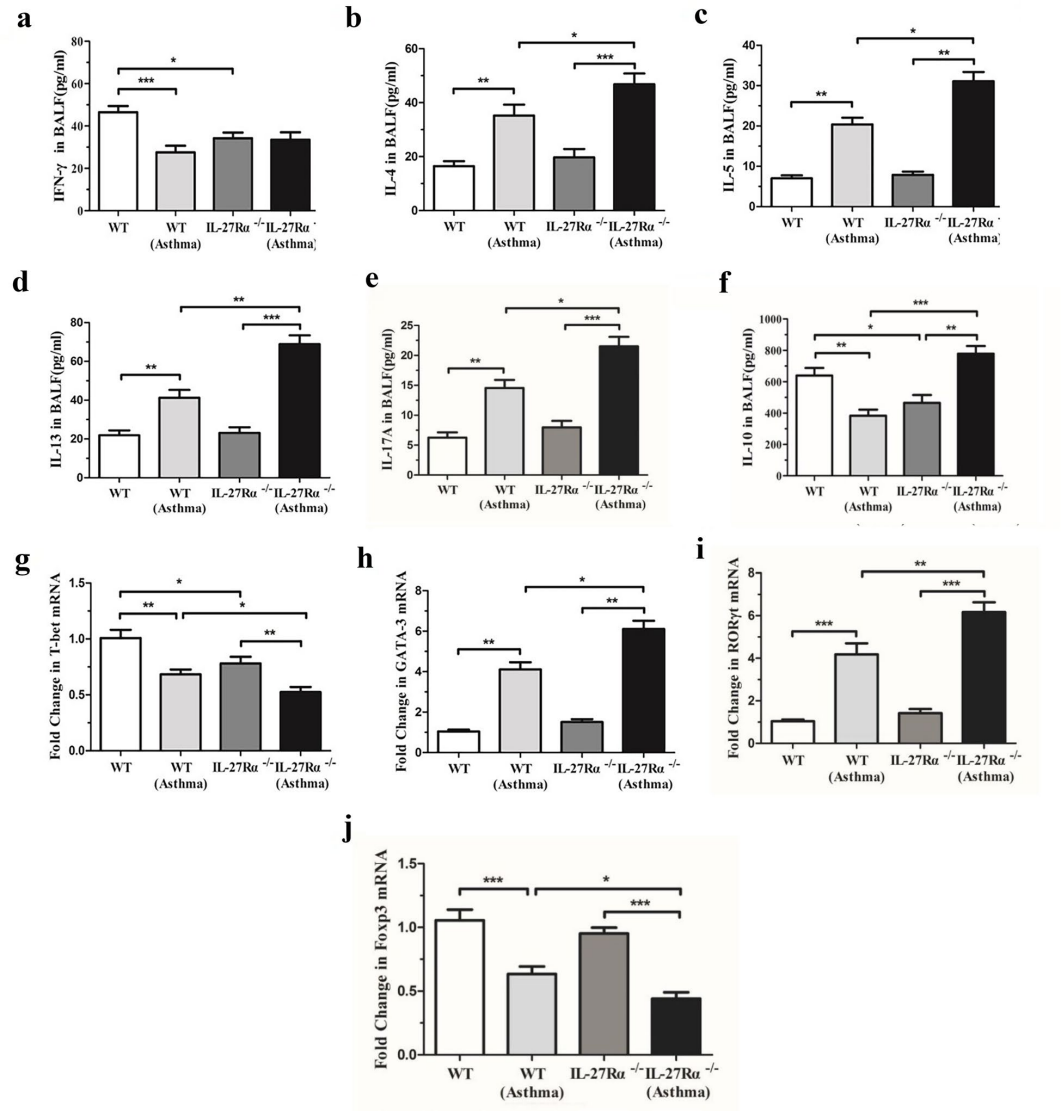
Effect of IL-27Rα gene knockout on Th cell-related factors. **a** The effect of IL-27Rα gene knockout on Th1 cell-related factor IFN-γ in BALF of asthmatic mice; **b**, **c** and **d **Effects of IL-27Rα gene knockout on Th2 cell-related factors IL-4, IL-5 and IL-13 in BALF of asthmatic mice; **e** The effect of IL-27Rα gene knockout on Th17 cell-related factor IL-17A in BALF of asthmatic mice; **f** The effect of IL-27Rα gene knockout on Treg cell-related factor IL-10 in BALF of asthmatic mice; **g** The effect of IL-27Rα gene knockout on Th1 cell transcription factor T-bet in lung tissue of asthmatic mice; **h** Effect of IL-27Rα gene knockout on Th2 cell transcription factor GATA-3 in lung tissue of asthmatic mice; **i** Effect of IL-27Rα gene knockout on Th17 cell transcription factor RORγt in lung tissue of asthmatic mice; **j** Effect of IL-27Rα gene knockout on Treg cell transcription factor Foxp3 in lung tissue of asthmatic mice. * *P* < 0.05, * * *P* < 0.01, * * * *P* < 0.001.

The results showed that compared with WT normal mice, the levels of IFN-γ and IL-10 in BALF of IL-27Rα^−/−^normal mice were decreased (*P* < 0.05). The levels of IL-4, IL-5, IL-13, and IL-17 A in BALF of WT asthmatic mice were higher than those of WT normal mice, while the levels of IL-10 and IFN-γ were lower (*P* < 0.05), and the levels of IL-4, IL-5, IL-13, and IL-17 A were significantly increased (*P* < 0.05 or *P* < 0.01). It is worth noting that the content of IL-10 in BALF of IL-27Rα^−/−^ asthmatic mice was also significantly higher than that of WT asthmatic mice, and the difference between the groups was statistically significant (*P* < 0.001) (Fig. 5a to f).

The results showed that compared with WT normal mice, the expression of T-bet in lung tissue of IL-27Rα^−/−^normal mice was decreased (*P* < 0.05). The expression levels of GATA-3 and RORγt in lung tissue of WT asthmatic mice were higher than those of WT normal mice, while the expression of T-bet and Foxp3 decreased, and the difference between the groups was statistically significant (*P* < 0.01). Compared with WT asthmatic mice, the expression levels of GATA-3 and RORγt in lung tissue of IL-27Rα^−^/^−^asthmatic mice were increased (*P* < 0.05), while the expression levels of T-bet and Foxp3 were decreased (*P* < 0.05) (Fig. 5g to j).

### IL-27Rα Gene =Knockout Promotes the Expression of NLRP3 Inflammasome

**Fig. 6:**
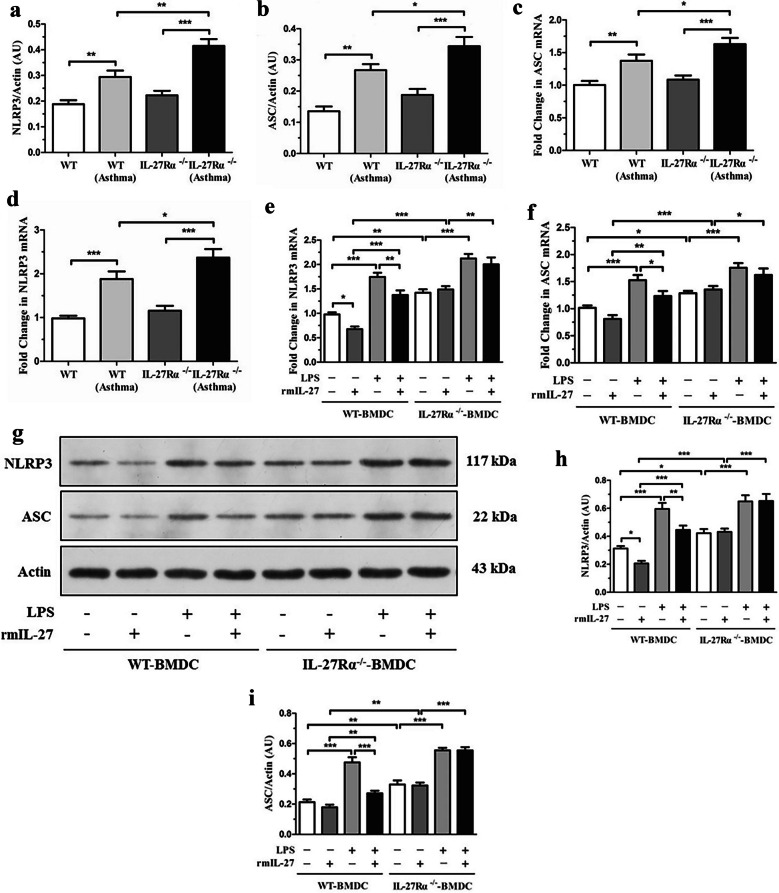
Effect of IL-27Rα gene knockout on the expression of NLRP3 inflammasome asthmatic mice. **a** Western blot was used to detect the expression of NLRP3 protein in lung tissue of mice in each group; **b** Western blot was used to detect the expression of ASC protein in lung tissue of mice in each group. **c** and **d** qPCR was used to detect the expression levels of NLRP3 and ASC mRNA in lung tissues of mice in each group. Effect of IL-27Rα gene knockout on the expression of NLRP3 inflammasome in BMDC. **e** and **f** qPCR was used to detect the mRNA expression levels of NLRP3 and ASC in WT-BMDC and IL-27Rα-/-BMDC after 48 h of rmIL-27 or/and LPS intervention; **g**, **h** and **i** Western blot was used to detect the expression levels of NLRP3 and ASC proteins in WT-BMDC and IL-27Rα-/-BMDC after 48 h of rmIL-27 or/and LPS intervention; * *P* < 0.05, * * *P* < 0.01, * * * *P* < 0.001.* *P* < 0.05, * * *P* < 0.01, * * * *P* < 0.001.

The results showed that there was no significant difference in the expression levels of NLRP3 and ASC mRNA and protein in lung tissue of IL-27Rα^−/−^normal mice compared with WT normal mice (*P* > 0.05) (Fig. 6a and b). The mRNA and protein expression levels of NLRP3 and ASC in lung tissue of WT asthmatic mice were significantly higher than those of WT normal mice (*P* < 0.01 or *P* < 0.001) (Fig. 6c and d).

The expression level of Caspase-1 in lung tissue of WT asthmatic mice was higher than that of WT normal mice but lower than that of IL-27Rα^−/−^asthmatic mice, and the difference between groups was statistically significant (*P* < 0.05) (Supplementary information, Fig. 3)*.* Our research further verified that compared with WT asthmatic mice, the expression levels of IL-1β and IL-18 protein in lung tissues of IL-27Rα^−/−^asthmatic mice were further increased, and the difference between groups was statistically significant (*P* < 0.05) (Supplementary information 2, Fig. 4).

There was no significant difference in the expression of NLRP3 between IL-27Rα^−/−^BMDC control group and IL-27Rα^−/−^BMDC rmIL-27 group (*P* > 0.05), but it was significantly higher than that of WT-BMDC control group and WT-BMDC rmIL-27 group (*P* < 0.01). The expression levels of NLRP3 mRNA and protein in the IL-27Rα^−/−^BMDC LPS group were significantly higher than those in IL-27Rα^−/−^BMDC control group (*P* < 0.001), but there was no significant difference between IL-27Rα^−/−^BMDC rmIL-27^+^ LPS group (*P* > 0.05) (Fig. 6e, g and h). The expression levels of ASC mRNA and protein in the IL-27Rα^−/−^BMDC control group were higher than those in the WT-BMDC control group (*P* < 0.05 or *P* < 0.01). The expression levels of ASC mRNA and protein in the IL-27Rα^−/−^BMDC rmIL-27 group were also higher than those in the WT-BMDC rmIL-27 group (*P* < 0.001 or *P* < 0.01) (Fig. 6f, g, and i). Compared with WT asthmatic mice, the expression levels of IL-1β and IL-18 protein the supernatant of BMDC culture medium of IL-27Rα^−/−^asthmatic mice were increased, and the difference between groups was statistically significant (*P* < 0.05) (Supplementary information 2, Fig. 5).

### IL-27Rα Gene Knockout Reduces the Expression of CD39

**Fig. 7:**
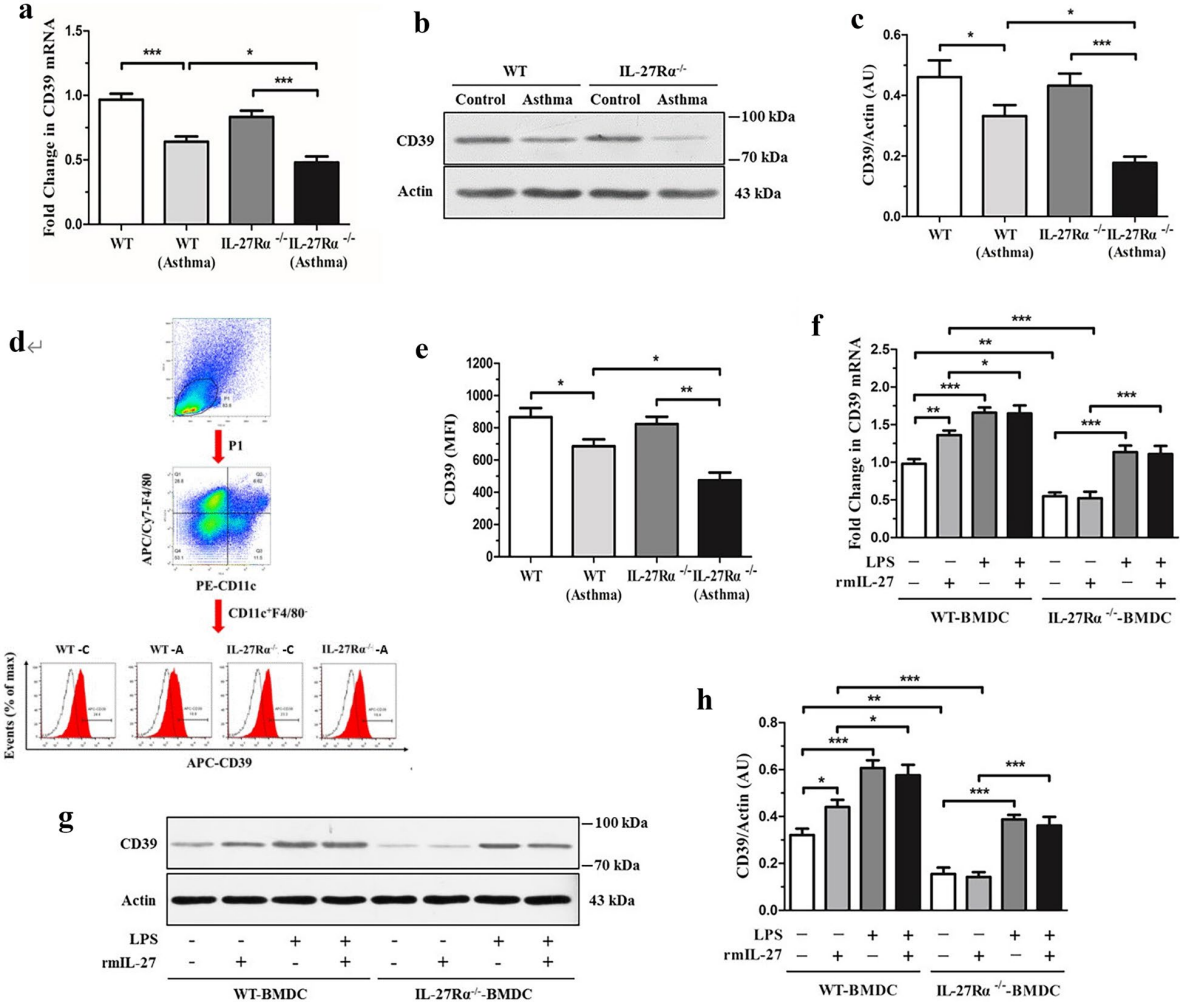
Effects of IL-27Rα gene knockout on the expression of CD39 mRNA. **a** qPCR was used to detect the expression of CD39 mRNA in the lung tissue of mice in each group. **b** and **c** Western blot was used to detect the expression of CD39 protein in the lung tissue of mice in each group. **d** Flow cytometry to detect the expression of CD39 in mouse lung dendritic cells flow diagram; **e** The effect of IL-27Rα gene knockout on the expression of CD39 mean fluorescence intensity (MFI) in lung dendritic cells of asthmatic mice. **f** qPCR was used to detect the expression of CD39 mRNA in WT-BMDC and IL-27Rα-/-BMDC after rmIL-27 or/and LPS intervention; **g** and **h** Western blot was used to detect the expression of CD39 protein in WT-BMDC and IL-27Rα-/-BMDC after rmIL-27 or (and) LPS intervention. * *P* < 0.05, * * *P* < 0.01, * * * *P* < 0.001.* *P* < 0.05, * * *P* < 0.01.* *P* < 0.05, * * * *P* < 0.001.

The results of qPCR confirmed that compared with WT normal mice, the expression level of CD39 mRNA in lung tissue of IL-27Rα^−/−^normal mice tended to decrease, but the difference was not statistically significant (*P* > 0.05). The expression level of CD39 mRNA in lung tissue of WT asthmatic mice was lower than that of WT normal mice (*P* < 0.001), while the expression level of CD39 mRNA in lung tissue of IL-27Rα^−/−^asthmatic mice was significantly lower than that of IL-27Rα^−/−^normal mice and WT asthmatic mice (*P* < 0.001 and *P* < 0.05) (Fig. 7a). The results of Western blot also confirmed that the expression level of CD39 protein in lung tissue of WT asthmatic mice and IL-27Rα^−/−^asthmatic mice was lower than that of WT normal mice and IL-27Rα^−/−^normal mice (*P* < 0.05 and *P* < 0.001), respectively. Compared with WT asthmatic mice, the expression level of CD39 protein in lung tissue of IL-27Rα^−/−^asthmatic mice was further decreased, and the difference between groups was statistically significant (*P* < 0.05) (Fig. 7b and c).

The results showed that there was no significant difference in the mean fluorescence intensity (MFI) of CD39 expression in lung dendritic cells (CD11c ^+^ F4/80-cell population) between WT normal mice and IL-27Rα^−/−^normal mice. Compared with WT normal mice, the MFI value of CD39 in lung dendritic cells of WT asthmatic mice was significantly decreased (*P* < 0.05), while the expression level of CD39 in lung dendritic cells of IL-27Rα^−/−^asthmatic mice was further decreased compared with WT asthmatic mice, and the difference was statistically significant (*P* < 0.05) (Fig. 7d and e).

The results of qPCR showed that the expression level of CD39 in the IL-27Rα^−/−^BMDC control group was lower than that in the WT-BMDC control group (*P* < 0.01). Compared with WT-BMDC control group, the expression of CD39 in WT-BMDC rmIL-27 group and WT-BMDC LPS group was increased (*P* < 0.01 or *P* < 0.001), but in IL-27Rα^−/−^BMDC group, only the expression of CD39 in LPS group was increased (*P* < 0.001), while there was no significant difference between rmIL-27 group and control group (*P* > 0.05). The results of the Western blot also confirmed that under normal conditions, the expression level of CD39 in WT-BMDC was higher than that in IL-27Rα^−/−^BMDC (*P* < 0.01). rmIL-27 or LPS intervention could increase the expression level of CD39 in WT-BMDC, and the difference between groups was statistically significant (*P* < 0.05 or *P* < 0.001) (Fig. 7f to h).

### IL-27Rα Gene Knockout Increases the Expression of ATP in BALF and BMDC

**Fig. 8:**
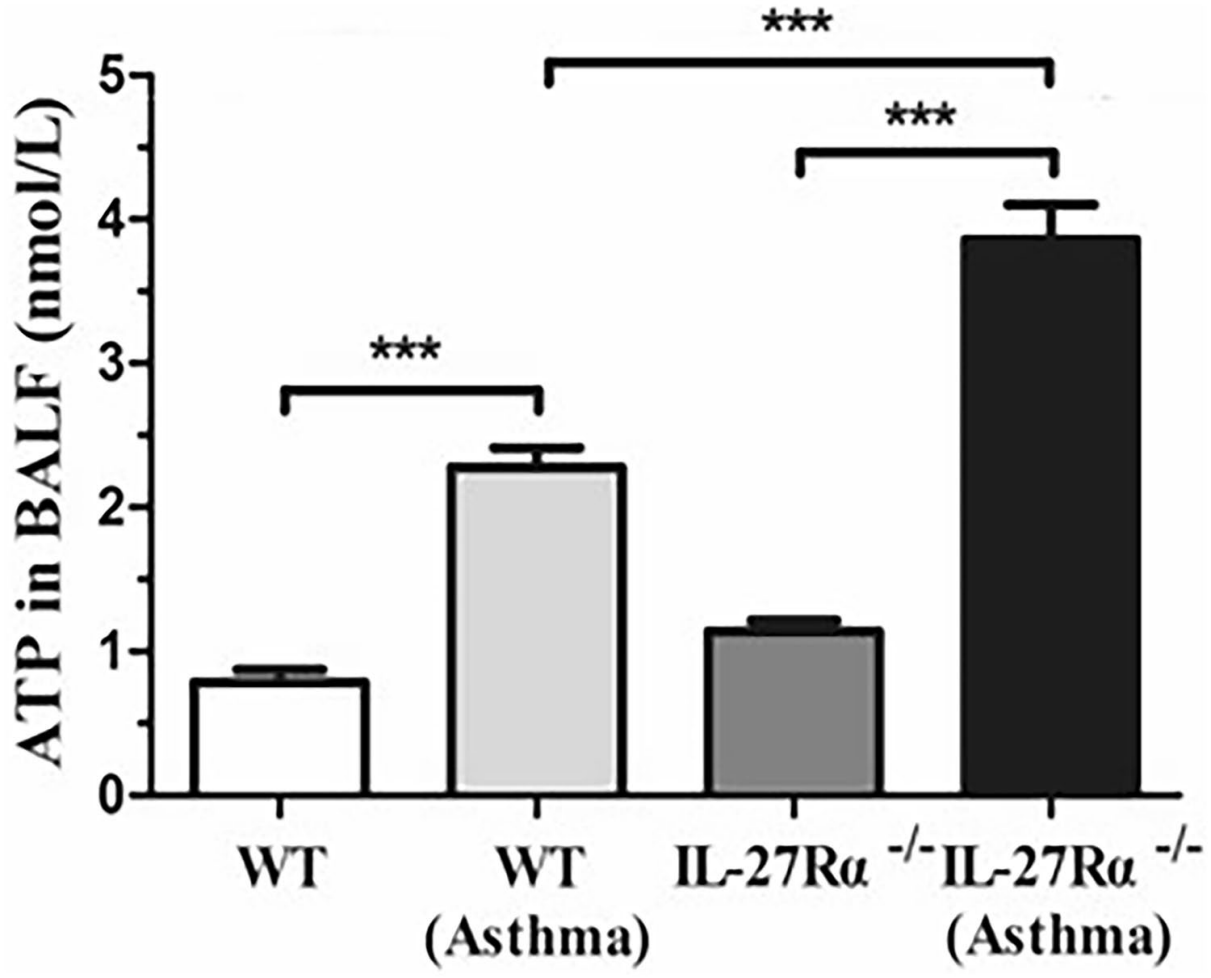
Effect of IL-27Rα gene knockout on ATP content in bronchoalveolar lavage fluid (BALF) of asthmatic mice. * * * *P* < 0.001.

The results showed that the ATP content in BALF of IL-27Rα^−/−^normal mice was higher than that of WT normal mice, but there was no significant difference between the groups (*P* > 0.05). Compared with WT normal mice, ATP content in BALF of WT asthmatic mice was significantly increased, while ATP content in BALF of IL-27Rα^−/−^asthmatic mice was higher than that of WT asthmatic mice, and the differences between groups were statistically significant (*P* < 0.001) (Fig. 8). Our study further verified the effect of IL-27Rα gene knockout on the ATP hydrolysis function of mouse BMDC. The results showed that the ATP content in the supernatant of the IL-27Rα^−/−^BMDC rmIL-27 group was also significantly higher than that in the WT-BMDC rmIL-27 group (95.333 ± 7.579 μmol/L vs.16.713 ± 3.888 μmol/L), and the difference was statistically significant (*P* < 0.001) (Supplementary information 2, Fig. 6).

### The Role of the JAK/STAT Signaling Pathway in the Regulation of BMDC CD39 Expression by IL-27

Among the phosphorylated (p-) JAK family members, the expression of p-JAK1 and p-JAK2 proteins in WT-BMDCs showed significant changes within 60 min after rmIL-27 intervention: In the initial 15 min, the expression levels of p-JAK1 and p-JAK2 proteins in WT-BMDCs were significantly higher than those before rmIL-27 intervention (*P* < 0.001), and the p-JAK1/JAK1 ratio increased and remained at a high level for the next 45 min. Since rmIL-27 promoted the simultaneous increase of JAK2 expression, the p-JAK2/JAK2 ratio did not change significantly. The above phenomenon was not observed in IL-27Rα^−^/^−^BMDC after rmIL-27 intervention, and the expression of p-JAK1 and p-JAK2 in IL-27Rα- / -BMDC remained at a low level.

After rmIL-27 intervention, the expression level of WT-BMDC STAT group members increased or decreased within a certain range, but there was no statistical difference (*P* > 0.05), while IL-27Rα^−^/^−^BMDC was hardly affected by rmIL-27. Among the phosphorylated STAT family members, the expression levels of p-STAT1 and p-STAT3 in WT-BMDCs after rmIL-27 intervention were significantly higher than those before intervention (*P* < 0.05) (Supplementary information 2, Fig. 7).

## DISCUSSION

At present, animal experiments have confirmed the role of IL-27 in inhibiting the pathogenesis of asthma, but different clinical studies have reported different levels of IL-27 expression in asthma patients. This may be closely related to the different immune response intensities of different disease individuals after allergen stimulation. It is also affected by multiple factors such as allergen type, time limit of hypersensitivity reaction, disease course, and research object (tissue/cell). The inhibitory effect of IL-27 on airway inflammation and airway hyperresponsiveness in asthma may be the combined effect of different types of Th cells regulated by IL-27. IL-27 inhibits Th2 cell differentiation and reduces the secretion of cytokines such as IL-4, IL-5, and IL-13, which are critical in promoting IgE synthesis, inducing eosinophil chemotaxis and recruitment, and increasing airway mucus secretion (mainly Muc5AC protein). [5–7] At the same time, IL-27 can inhibit Th17 cell differentiation and IL-17 secretion by down-regulating the expression levels of RORα and RORγ in Th0 cells, and reduce the release of pro-inflammatory factors such as IL-6, IL-8, and prostaglandin (PG) E2 from airway epithelial cells, fibroblasts, and stromal cells, and exert an inhibitory effect on Th17 immune response. [8] It is worth noting that our study confirmed that the contents of IFN-γ and IL-10 in BALF of IL-27Rα^−^/^−^normal mice were lower than those of WT asthmatic mice, but the content of IL-10 in BALF of IL-27Rα^−^/^−^asthmatic mice was significantly higher than that of WT asthmatic mice, and IFN-γ also showed an increasing trend [9].

Although basic studies have confirmed that, on the one hand, IL-27 not only promotes T-bet expression by inducing STAT1 phosphorylation but also up-regulates the expression level of surface molecule IL-12Rβ2 and increases IL-12-dependent IFN-γ synthesis, on the other hand. IL-27 can promote the expression of early growth response gene (Egr) -2 and B lymphocyte-induced maturation protein (Blimp) -1 in Tr1 cells through STAT1 and STAT3 signaling pathways, and play a positive regulatory role in the synthesis of IL-10. IL-27 may cooperate with other factors to promote the differentiation of Th1 and Tr1 cells, induce the synthesis of IFN-γ and IL-10, and directly or indirectly inhibit the excessive immune response of the body. In addition, for the non-corresponding situation the content of IFN-γ and IL-10 in BALF of IL-27Rα^−^/^−^asthmatic mice increased (or tended to increase), while the expression of T-bet and Foxp3 in lung tissue decreased, we speculated that the causes include: (1) IL-27 may regulate Th cell differentiation and cytokine synthesis through different mechanisms, and there are differences in the regulatory pathways between the two; (2) IL-27 may regulate Th cell differentiation and cytokine synthesis through different mechanisms. (2) The cells that produce IFN-γ and IL-10 are not limited to Th1, Treg and Tr1 cells. Recent studies have confirmed that innate lymphoid cells (ILC) are also an important source of these factors Current studies have shown that IL-27, as an immunomodulatory factor, inhibits airway inflammation and airway hyperresponsiveness in asthma. Most studies have confirmed that this is related to the differential regulation of Th cells by IL-27. However, asthma is a chronic inflammatory airway disease involving multiple cells and cellular components. In addition to T cells, IL-27 may also regulate other types of cells such as dendritic cells, macrophages, and natural killer cells (NK) [10, 11].

In this study, we first confirmed that the expression level of CD39 in the lung tissue of asthmatic mice was lower than that of normal control mice, while the ATP content in BALF was significantly higher than that of normal control mice. Secondly, we found that although there was no significant difference in the expression level of CD39 in the lung tissue between WT normal mice and IL-27Rα^−^/^−^normal mice, the expression level of CD39 in lung tissue of IL-27Rα^−^/^−^asthmatic mice was significantly lower than that of WT asthmatic mice. Correspondingly, the ATP content in BALF of IL-27Rα^−^/^−^asthmatic mice was significantly higher than that of WT asthmatic mice, but there was no significant difference between WT mice and IL-27Rα^−^/^−^mice under normal conditions. Therefore, it is speculated that the increase of airway responsiveness and airway inflammation in asthmatic mice caused by IL-27Rα gene knockout may be closely related to the decrease of CD39 expression and the increase of ATP content in the airway of asthmatic mice. The dysfunction of IL-27 may be an important factor leading to the imbalance of the CD39/ATP axis in the lung tissue of asthmatic mice [12].

NLRP3 inflammasome is involved in airway inflammation. Studies have confirmed that compared with WT asthmatic mice, NLRP3 gene knockout (Nlrp3^−^/^−^), ASC/PYCARD gene knockout (Pycard^−/−^), or Caspase-1 gene knockout (Caspase-1^−^/^−^) asthmatic mice established by OVA sensitization and challenge model have significantly reduced inflammatory cell infiltration in lung tissue, serum IgE level and Th2 and Th17 cytokine content in BALF, indicating that NLRP3 inflammasome is essential in the occurrence or aggravation of asthmatic airway inflammation. In addition, Lee TH et al. proposed that neutrophilic airway inflammation is inseparable from the activation of NLRP3 inflammasome. IL-1β produced after the activation of NLRP3 inflammasome positively regulates the differentiation of Th17 cells, which promotes the secretion of IL-6 and IL-8 and induces neutrophil recruitment, which may be an important factor in the acute attack of asthma. Our study confirmed that IL-27Rα gene knockout can lead to an imbalance of CD39/ATP axis function in lung tissue of asthmatic mice: IL-27 dysfunction leads to insufficient expression of CD39, and excessive ATP activates downstream NLRP3 inflammasome, which induces inflammatory waterfall effect in the lesion microenvironment through IL-1β and IL-18, and promotes Th2 and Th17 immune response bias [13].

We speculate that the possible mechanism is that there are a large number of endogenous ' danger signals ' that can activate NLRP3 inflammasome in the airway of mice during acute asthma attacks. The most representative one is that allergens induce a large amount of ATP released by a variety of immune cells and inflammatory cells. IL-27Rα gene knockout leads to decreased CD39 expression or limited hydrolysis function of some specific/non-specific cells in the lung tissue of asthmatic mice, and a large amount of ATP is accumulated in the internal environment. The P2X7 receptor induces the assembly and activation of NLRP3 inflammasome components NLRP3, ASC, and Pro-caspase-1 to form Caspase-1 with enzymatic cleavage function. The latter acts on the precursor of IL-1 family members to make them active mature IL-1β and IL-18, both of which are released into the extracellular to promote Th2 and Th17 immune responses. Finally, the airway reactivity of asthmatic mice was increased and the airway inflammatory characteristics were aggravated [14].

Dendritic cells are an important source of IL-27 production. Previous studies have focused on the effects of IL-27 derived from dendritic cells on T cell proliferation, differentiation, and secretion in different disease environments. It has been confirmed that dendritic cells express IL-27 receptors at the same time, and the synthesized IL-27 has an ‘autocrine effect’ on dendritic cells themselves. Visperas A et al. found that in the mouse colitis model, IL-27 acts on dendritic cells and enhances the ability of LPS to induce IL-6 and IL-1β synthesis and secretion, and plays a positive regulatory role in Th17 cell differentiation and Th17 immune response, eventually causing explosive colitis. Mascanfroni ID et al. [15] confirmed that the synthesis of IL-6, IL-12, and IL-23 in dendritic cells from the spleen of IL-27Rα^−^/^−^mice derived from experimental autoimmune encephalomyelitis (EAE) increased, while the secretion of IL-10 decreased, which promoted Th1 and Th17 immune responses and aggravated the infiltration of inflammatory cells in the central nervous system. So, in the asthma disease environment, whether IL-27 has a similar ‘autocrine’ regulatory effect on dendritic cells is rarely reported in the relevant literature.

## CONCLUSION

IL-27Rα gene knockout leads to increased airway hyperresponsiveness and airway inflammation in asthmatic mice. The possible mechanism is that IL-27 dysfunction causes decreased CD39 expression and limited ATP hydrolysis function of dendritic cells in asthmatic mice through JAK (1/2)/STAT (1/3) signaling pathway. The imbalance of the CD39/ATP axis leads to excessive activation of downstream NLRP3 inflammasome, releases a large number of pro-inflammatory factors such as IL-1β and IL-18, aggravates the polarization of Th2 and Th17 cells, and ultimately promotes airway inflammation and airway hyperresponsiveness in asthma.

### SUPPLEMENTARY INFORMATION

Below is the link to the electronic supplementary material.

Supplementary file1 (DOCX 58 KB)

Supplementary file2 (DOCX 37988 KB)

## Data Availability

All authors had full access to all of the data in the study and takes responsibility for the integrity of the data and the accuracy of the data analysis, including and especially any adverse effects.
