# Supplementary material for: IL-27 Alleviates Airway Inflammation and Airway Hyperresponsiveness in Asthmatic Mice by Targeting the CD39/ATP Axis of Dendritic Cells
Source: Inflammation. 2023 Dec 20;47(2):807–21. doi: 10.1007/s10753-023-01945-9 (PMC11074049; doi:10.1007/s10753-023-01945-9)
Supplement: Supplementary file 1 — Supplementary file1 (DOCX 58 KB) [file 10753_2023_1945_MOESM1_ESM.docx]

**Experimental Method**

**1. Genotype identification of IL-27Rαknockout mice**

(1) Extraction of mouse genomic DNA: About 0.5 cm of mouse tail tip was cut and 180 μL sample lysis buffer A was added. 20 μl proteinase K was added, and the mixture was fully vortexed and mixed. The mixture was incubated in a water bath at 55 °C until complete lysis. During the incubation period, the sample can be occasionally taken out for vortexing to accelerate the cracking rate. It usually takes 6 ~ 8 hours to crack completely. For convenience, it can be directly cracked overnight. Each sample needs to be mixed with 200 μl sample pyrolysis solution B and 200 μl absolute ethanol. According to this ratio, the appropriate volume of sample pyrolysis liquid B and anhydrous ethanol were mixed and vortexed. The sample tube was violently vortexed at the highest speed for 15 seconds, and then the equal volume mixture of the sample pyrolysis liquid B and anhydrous ethanol was added, 400 μl / tube, violently vortexed, and mixed. After adding the equal volume mixture of sample pyrolysis solution B and anhydrous ethanol, the sample tube may produce a white precipitate. The purification column was placed on the waste liquid collection tube, and all the obtained mixture was added to the DNA purification column. After high-speed centrifugation (> 6000 g × 1 min), the waste liquid was discarded and the waste liquid collection tube was recovered. Note: The precipitate must be transferred to the DNA purification column, otherwise it will seriously affect the extraction effect. The purification column was placed on the waste liquid collection tube, 500 μl of detergent I was added, and high-speed centrifugation (> 6000 g × 1 min) was performed. Discard the waste liquid to collect the liquid in the pipe. The purification column was placed on the waste liquid collection tube, 600 μl of detergent II was added, and high-speed centrifugation (> 18000 g × 1 min) was performed. Discard the waste liquid to collect the liquid in the pipe. Repeated high-speed centrifugation (> 18000 g × 1 min) to remove residual ethanol. The DNA purification column was placed on a clean 1.5 ml centrifuge tube, 50 ~ 200 μl eluent was added, placed at room temperature for 1 ~ 3 minutes, and finally centrifuged at a high speed (> 18000 g × 1 min). The obtained liquid was the total DNA of the purified tissue.

(2) PCR reaction: Primers were synthesized according to the identification data provided by Jackson Laboratory (http://jaxmice.jax.org/strain/018078.html). The specific primer sequences were as follows:

| Gene name | Primer sequences | | Product（bp） |
| --- | --- | --- | --- |
| IL-27 wild | Forward 5’-CAAGACCTTGTGTGCAGGTG-3’ | | 397 bp |
|  | Reversed 5’-GTCACCATCTTGAGCCCAGT-3’ | |  |
| IL-27 mutant | Forward 5’-CTTGGGTGGAGAGGCTATTC-3’ | | 280 bp |
|  | Reversed 5’-AGGTGAGATGACAGGAGATC-3’ | |  |
| CD39 | Forward | 5’-CATCCAAGCATCACCAGACT-3’ | 154 |
|  | Reversed | 5’-ATGATCTTGGCACCCTGGAA-3’ |  |
| NLRP3 | Forward | 5’-ACCAGCCAGAGTGGAATGAC-3’ | 112 |
|  | Reversed | 5’-ATGGAGATGCGGGAGAGATA-3’ |  |
| ASC | Forward | 5’-CACCAGCCAAGACAAGATGA-3’ | 121 |
|  | Reversed | 5’-CTCCAGGTCCATCACCAAGT-3’ |  |
| IL-1β | Forward | 5’-AGTTGACGGACCCCAAAAG-3’ | 145 |
|  | Reversed | 5’-CTTCTCCACAGCCACAATGA-3’ |  |
| IL-18 | Forward | 5’-TGGAGACCTGGAATCAGACA-3’ | 147 |
|  | Reversed | 5’-TGGGGTTCACTGGCACTT-3’ |  |

Construction of PCR reaction system: Genotyping was performed using the improved PCR reaction system (Refer to the Tiangen Biochemical Technology Co., Ltd. PCR kit instructions). The corresponding components were added to the 200 μl PCR tube according to the following ratio, mixed, and quickly centrifuged.

| 2×Taq PCR Master Mix | 7.5 µl |
| --- | --- |
| Forward Primer（10 µmol/L） | 0.6 µl |
| Reverse Primer（10 µmol/L） | 0.6 µl |
| cDNA template | 1 µl |
| dH_2_O | 5.3 µl |
| Total | 15 µl |

(3) Electrophoresis and expected results: After the PCR reaction, the above PCR products were subjected to electrophoresis with 2 % agarose gel, and analyzed by an automatic gel imaging system. The product size was determined according to DNA Marker. The expected product size: wild type (WT): 397 bp, IL-27Rα^- / -^homozygote (Mutant): 280 bp, IL-27Rα- / -heterozygote (Heterozygote): 280 bp and 397 bp.

**2. Airway hyper-responsiveness (AHR) was measured**

Twenty-four hours after the last challenge, the airway hyperresponsiveness of mice in each group was evaluated by invasive method using BUXCO small animal lung function instrument (Fine Pointe TM series). The specific detection methods are as follows: 1 % pentobarbital sodium was injected intraperitoneally to anesthetize mice (60 mg / kg body weight of mice). After satisfactory anesthesia, tracheal intubation was performed in the anterior cervical region and connected to a micro-ventilator. The respiratory rate was set to 140 ~ 150 bpm, and the flow rate was adjusted to 0.13 ~ 0.15 ml of tidal volume in mice. By detecting the changes of airway airflow and pressure in mice, the instrument automatically calculates the parameters such as airway resistance index (RI) and dynamic lung compliance (Cdyn). After successful setting, the basic value of airway resistance in mice was recorded (the resistance during PBS atomization was the basic value), and then the changes of airway resistance and dynamic lung compliance in mice after twice increasing methacholine (Mch) atomization were measured. Mch atomization concentration was set to 6 gradients, from low too high in order of 0,3.125,6.25,12.5,25,50 mg / ml. Each Mch atomization time was set to 30 seconds, the recording time was 3 minutes, and the time to return to baseline was 4 minutes.

**3. Bronchoalveolar lavage**

The mice were lightly anesthetized by intraperitoneal injection of 1 % pentobarbital sodium (about 40 mg / kg body weight of mice), and then blood was taken from the eyeball. After blood collection, the mice were sacrificed by cervical dislocation, and bronchoalveolar lavage was performed with sterile PBS buffer containing 1 mmol / L EDTA. During lavage, the neck was dissected first, and the pre-tracheal tissue of the neck was carefully separated and removed. The trachea was fully exposed, and a 24GY venous indwelling needle was inserted. The needle core was removed and the needle tube was fixed with the surgical line to prevent shedding. The other end of the needle tube was connected to a 1 ml syringe containing sterile lavage fluid. The amount of liquid for each alveolar lavage was 0.5ml, and repeated suction was performed each time for a total of three times. The recovery rate of bronchial alveolar lavage fluid (BALF) was more than 80 % ( recovery rate = recovery volume / lavage volume × 100 % ).

**4. Wright-Giemsa (Wright-Giemsa) staining**

The collected bronchoalveolar lavage fluid was centrifuged at low temperature ( 1200 r / min × 7 min, 4 ° C ), and the supernatant was collected and stored at − 80 ° C for the detection of cytokines such as IL-4, IL-5 and IL-13. The cell precipitate after centrifugation was observed. If the red color was visible to the naked eye, an appropriate amount ( 100-200 μl ) of red blood cell lysate was added to break the red color. After full blowing, the cells were allowed to stand at room temperature for 7 minutes. Then 2 ml of PBS buffer containing 1 % fetal bovine serum (FBS) was added for dilution and termination of the reaction. The cells were centrifuged at low temperature (1200 r / min × 7 min, 4 ° C), and the supernatant was discarded (selected as a step). Then, 500 μl of PBS buffer containing 1 % FBS was resuspended, and 10 μl of cell suspension was taken for counting. Finally, the cells in each group were adjusted to a consistent concentration (about 2 × 106 / ml). After the concentration was adjusted, 100 μl cell suspension was added to the cell smear centrifuge, and the cell slides were prepared by centrifugation at 500 r / min × 1 min. Wright-Giemsa staining was performed after the cells were slightly dried.

The Wright-Giemsa staining kit contains two reagents, A and B. The A solution is mainly a methanol fixative, and the B solution is a Wright-Giemsa composite staining solution. The operation was carried out according to the kit instructions. The specific steps included : the slides containing cells were naturally dried at room temperature after the slides were shaken, and the circle marking was performed in the cell concentration area on the back of the slides ; take a drop of A droplet on the cell concentration area ( marked area ), and stand at room temperature for 1 minute ; after 1 minute, do not wash, directly take B droplets on the cell concentration area ( marked area ), and stand at room temperature for 8 minutes. Among them, the volume of liquid B is 2 times that of liquid A, and is added dropwise at this ratio. After 8 minutes, the slides were washed in running water for 30 ~ 60 seconds, and the direction and angle of washing were noted. After washing, the slides were dried naturally, observed under the microscope and classified and counted. In the microscopic field of view, according to the color of various particles in the cytoplasm and the morphological characteristics of the nucleus, BALF cells can be divided into mononuclear macrophages (Mac), eosinophils (Eos), neutrophils ( Neu ) and lymphocytes ( Lym ). The specific performance is that the Mac cell body is the largest, the cytoplasm is light purple or blue purple, and the nucleus is round or oval; the cytoplasm of Eos cells contained coarse orange-red granules, and the nucleus was mostly 2 leaves. The cytoplasm of Neu cells was purplish red, and the nucleus was lobulated (2-5 leaves). The Lym cells have small cell bodies, large nuclei, and light blue cytoplasm.

**5. Histopathological examination**

(1) Preparation of paraffin sections: 1) Sampling and fixation: The mouse lung tissue was fixed in 4 % neutral paraformaldehyde solution for more than 24 hours, so that the protein was fully denatured and solidified to maintain the complete morphological structure of the cells. 2) Conventional gradient dehydration: Generally, gradient alcohol (from low concentration to high concentration ) is used as dehydrating agent to gradually remove water from tissues. The specific concentration and time: 50 % ethanol 6 ~ 8 hours, 70 % ethanol 3 ~ 4 hours, 85 % ethanol 3 ~ 4 hours, 95 % ethanol I liquid 1 hour, 95 % ethanol II liquid 1 hour, 100 % ethanol I liquid 30 minutes, 100 % ethanol II liquid 30 minutes. 3) Xylene transparent: Xylene as a transparent agent can replace alcohol in the tissue, the specific time: xylene I liquid 30 minutes, xylene II liquid 30 minutes. 4) Wax immersion and embedding: The lung tissue after xylene transparency was immersed in soft wax (melting point 45 ° C ~ 50 ° C ) for 1 hour, and hard wax ( melting point 56 ° C ~ 58 ° C ) for 1 hour. After that, the paraffin-immersed lung tissue was taken out and placed in a pre-heated mold. Pay attention to the placement direction of the tissue section, and then fill in the paraffin with the same melting point as the hard wax, and wait for the wax liquid to cool naturally. 5) Slicing, developing and sticking: The embedded wax blocks were taken out, the excess paraffin was cut off, fixed on the paraffin slicing machine, and cut into slices with a thickness of 5μm. Then, the tissue sections were put into hot water to develop the slices. After the slices were completely flattened, they were attached to the slides, marked, and dried in a 38 ° C incubator.

(2). Hematoxylin-eosin (HE) staining: dewaxing: xylene I solution for 10 minutes, xylene II solution for 10 minutes. Hydration: 100 % ethanol I solution for 5 minutes, 100 % ethanol II solution for 5 minutes, 95 % ethanol I solution for 5 minutes, 95 % ethanol II solution for 5 minutes, 85 % ethanol for 5 minutes, 75 % ethanol for 5 minutes, distilled water for 5 minutes. Dyeing: Hematoxylin was stained at room temperature for 5-10 minutes, and the excess dye was removed by running water for 30 seconds. At this time, the staining effect of the nucleus could be observed under a microscope. If dyed blue, available 1 % hydrochloric acid-alcohol differentiation 15 seconds, water rinse 1 minute, then 1 % dilute ammonia water back blue 30 seconds, water rinse 10 ~ 15 minutes (optional steps). Staining with 0.5 % eosin alcohol at room temperature for 1 ~ 3 minutes, and the staining effect was observed under a microscope. If the staining is too red, can use 85 % or 90 % ethanol appropriate differentiation (selected as a step). Dehydration transparent: 95 % ethanol I liquid 5 minutes, 95 % ethanol II liquid 5 minutes, 100 % ethanol I liquid 5 minutes, 100 % ethanol II liquid 5 minutes, xylene I liquid 10 minutes, xylene II liquid 10 minutes. Seal: Neutral gum seal.

(3) Periodic acid-schiff (PAS) staining: dewaxing: xylene I solution for 10 minutes, xylene II solution for 10 minutes. Hydration: 100 % ethanol I solution for 5 minutes, 100 % ethanol II solution for 5 minutes, 95 % ethanol I solution for 5 minutes, 95 % ethanol II solution for 5 minutes, 85 % ethanol for 5 minutes, 75 % ethanol for 5 minutes, distilled water for 5 minutes. Staining I: Periodic acid staining for 5 ~ 10 minutes, rinsed with running water for 10 minutes. Staining II: dyeing with schiff 's staining solution for 10 ~ 15 minutes, washing with running water for 10 minutes. Staining III: Hematoxylin immersion for 1 ~ 2 minutes, rinse with running water for 10 minutes. Dehydration transparent: 95 % ethanol I liquid 5 minutes, 95 % ethanol II liquid 5 minutes, 100 % ethanol I liquid 5 minutes, 100 % ethanol II liquid 5 minutes, xylene I liquid 10 minutes, xylene II liquid 10 minutes. Seal: Neutral gum seal.

(4). Observation and judgment of pathological results: Under light microscope (generally 200 times field of vision), 5 samples were selected from each group, and 5 representative fields of vision were selected from each sample to score the pathological changes of lung tissue inflammation (0 ~ 3 points). The specific scoring criteria are as follows: 0 points: There was almost no inflammatory cell infiltration around the bronchi and blood vessels in the lung tissue, the bronchial mucosal epithelium was intact, there was no inflammatory cell infiltration in the pulmonary interstitium, and the alveolar septum was intact. 1 point: Inflammatory cell infiltration was occasionally seen around the bronchi or blood vessels in the lung tissue, but no cell aggregation was observed. Bronchial mucosa was rarely detached. There was a little inflammatory cell infiltration in the pulmonary interstitium, and alveolar septum could be broken. 2 points: There were agglomerated inflammatory cells around the bronchus or blood vessels, and the number of cell layers was 1-5 layers. The bronchial mucosa fell off, and inflammatory cell infiltration was also observed in the pulmonary interstitium, and the alveolar septum was broken. 3 points: a large number of inflammatory cells infiltrated around the bronchus or blood vessels, and the aggregation was obvious. The number of cell layers was more than 5 layers. The bronchial mucosal epithelium was obviously exfoliated, and the interstitial lung was also infiltrated by agglomerated cells.Observation of PAS staining pathological results: Under light microscope (usually 200 times visual field), 5 samples were selected from each group, and 5 representative complete bronchiole cross sections (long diameter / short diameter < 2) were selected from each sample to observe the proliferation of goblet cells and mucus secretion in airway epithelium. Image Pro Plus 6.0 (IPP 6.0) software was used for image analysis, and airway basement membrane perimeter (Pbm, μm) and airway epithelial PAS positive area (APAS +, μm2) were measured. The positive area of PAS staining per unit length of basement membrane (APAS + / Pbm) was calculated.

**6. Immunohistochemical staining**

The basic steps of detecting the expression of Muc5AC protein in bronchial epithelium of lung tissue by immunohistochemistry: dewaxing: xylene I solution for 10 minutes, xylene II solution for 10 minutes. Hydration: 100 % ethanol I solution for 5 minutes, 100 % ethanol II solution for 5 minutes, 95 % ethanol I solution for 5 minutes, 95 % ethanol II solution for 5 minutes, 85 % ethanol for 5 minutes, 75 % ethanol for 5 minutes, distilled water for 5 minutes. Antigen repair: using microwave repair method. The tissue sections were placed in a repair box containing EDTA antigen repair solution (pH 6.0), heated to boil with high heat in a microwave oven, and then lowered the firepower to keep the liquid slightly boiled for 15 minutes. Pay attention to prevent excessive evaporation of antigen retrieval solution, do not dry. After natural cooling to room temperature, wash with PBS buffer 3 times, 5 minutes each time. Removal of endogenous peroxidase: The sections after antigen repair were placed in a wet box, and 3 % hydrogen peroxide solution (3 % H2O2) was added dropwise to cover the sections. The sections were incubated at room temperature for 20 minutes in dark, and then washed with PBS buffer for 3 times, 5 minutes each time. Closure: After the section is slightly dried, the immunohistochemical pen is used to mark the circle around the tissue to prevent the loss of antibodies. Add 3 % BSA solution and incubate for 30 minutes at room temperature or overnight at 4 °C. When closing, attention should be paid to maintaining humidity and preventing slices from drying. After the closure is completed, do not wash, carefully absorb the water around the slice with a filter paper. Incubation of primary antibody: Human anti-mouse Muc5AC antibody was diluted with antibody diluent (PBS buffer containing 3 % BSA) at a ratio of 1: 100, and carefully added to the section to completely cover the tissue. Incubation overnight at 4 °C. The next day, the samples were equilibrated at room temperature for 30 minutes, and then washed three times with PBS buffer for 5 minutes each time. Incubation of secondary antibody: HRP-labeled goat anti-human secondary antibody was carefully dropped onto the section to completely cover the tissue. The tissue was incubated at 37 °C for 30 minutes, and then washed three times with PBS buffer for 5 minutes each time. DAB color development: freshly prepared DAB color development solution according to the instructions of DAB color development kit, carefully added to the tissue section, and immediately observed under the microscope. The coloration time was controlled according to the degree of positive cell coloration (brownish yellow), and the coloration was terminated by flushing the sections with running water. Hematoxylin re-staining: Hematoxylin solution was used for re-staining at room temperature for 5 minutes, and the excess dye solution was removed by washing with running water for 30 seconds. Differentiate with 1 % hydrochloric acid-alcohol for several seconds, rinse with running water for 1 min, reverse blue with 1 % dilute ammonia for 30 seconds, and rinse with running water for 10-15 minutes. Dehydration, transparency, mounting: 95 % ethanol I solution for 5 minutes, 95 % ethanol II solution for 5 minutes, 100 % ethanol I solution for 5 minutes, 100 % ethanol II solution for 5 minutes, xylene I solution for 10 minutes, xylene II solution for 10 minutes. Finally, the neutral gum seal.

Observation and judgment of immunohistochemical results: Under light microscope (generally 200 times field of vision), 5 samples were selected from each group, and 5 representative cross-sections of bronchioles were selected from each sample to observe the location and intensity of Muc5AC protein expression positive (brownish yellow). IPP 6.0 software was used for image analysis to determine the average optical density (AOD) of Muc5AC protein expression intensity in airway epithelium and surrounding mucosa.

**7.Quantitative real-time PCR (qPCR) detection**

(1) Extraction of total RNA from lung tissue : 50 ~ 100 mg mouse lung tissue was placed in a glass homogenizer pretreated with 1 ‰ DEPC, and 1 ml TRIzol extract was added to fully grind the tissue. After that, the grinding fluid was transferred to a 1.5 ml enzyme-free tube. Stand at room temperature for 5 minutes to allow the nucleic acid to be fully lysed. Add 200μl chloroform (chloroform volume of 1 / 5 TRIzol extract) to the above 1.5ml enzyme-free tube, cover the lid, shake repeatedly for 15 seconds, and stand at room temperature for 5 minutes. The above mixture was centrifuged at low temperature and high speed (12000 g × 15 min, 4 ° C). After centrifugation, the sample was divided into three layers: RNA was present in the upper colorless aqueous phase, DNA and protein were present in the intermediate phase, and the lower layer was a red phenol-chloroform organic phase. Absorb the upper colorless water phase 500 ~ 600μl to the new 1.5ml enzyme-free tube, add an equal volume of isopropanol solution, cover the lid, turn up and down for 15 seconds, and stand at room temperature for 15 minutes. The above static mixture was centrifuged at low temperature and high speed (12000 g × 15 min, 4 ° C), and milky white adherent precipitation was observed after centrifugation. The supernatant was discarded, washed twice with 75 % ethanol solution (prepared with enzyme-free water), and centrifuged at low temperature (7500 g × 5 min, 4 ° C ). The supernatant was discarded, the tube cap was opened, and the RNA was naturally dried for 5 minutes and fully dissolved in 40-50μl enzyme-free water.

(2) Detection of RNA purity and concentration: The A260 and A280 values of the samples were detected by ultraviolet spectrophotometer, and the RNA purity was judged according to the A260 / A280 ratio. If the ratio is between 1.8 and 2.0, it can meet the experimental requirements. RNA concentration was calculated according to the formula: RNA concentration (ng / μl) = A260 value × dilution multiple × 40 ( ng / μl ). UV spectrophotometer to detect the specific steps of RNA purity: take 2 μl of RNA stock solution, add 48 μl of non-enzymatic water to dilute. The A260 and A280 values were set to zero by using non-enzymatic water as a blank control. The A260 and A280 values of the diluted samples were detected, and the A260 / A280 ratio and RNA concentration were calculated.

(3) Reverse transcription-polymerase chain reaction (RT-PCR): The complementary DNA (cDNA) was synthesized by RT-PCR. The specific steps are as follows: RNA thermal denaturation: 1.5ml non-enzymatic tube containing RNA was placed in a 65 ° C water bath for 5 minutes, and immediately cooled on ice after completion. RT-PCR reaction: Construct RT-PCR reaction system: Add the corresponding components of RT-PCR reaction to 200μl PCR tube according to the following proportion, mix well and centrifuge quickly.

| 5×RT Buffer | 2 µl |
| --- | --- |
| Enzyme Mix | 0.5 µl |
| Primer Mix | 0.5 µl |
| RNA | 0.5 pg~1 µg |
| Nuclease-free Water | up to 10 µl |
| Total volume | 10 µl |

RT-PCR reaction conditions:

37°C，15 min×1 cycle;

98°C，5 min×1 cycle;

4°C，forever;

(4) After the RT-PCR reaction, the PCR reaction can be performed immediately or the cDNA can be stored at − 20 ° C for later use.

(5) Primer design and synthesis of conventional PCR reaction : The mouse mRNA nucleotide sequence was obtained by NCBI gene bank ( Gene Bank, http://www.ncbi.nlm.gov/sites/ entrez ), and the primer design was performed using Primer 5.0 software. According to the primer design requirements, the appropriate primer sequence was selected for processing and synthesis. All primers in this experiment were synthesized by Shanghai Bioengineering Company. The specific sequences are as follows :

| Gene name | Primer sequences | | Product（bp） |
| --- | --- | --- | --- |
| T-bet | Forward | 5’-GGTGTCTGGGAAGCTGAGAG-3’ | 122 |
|  | Reversed | 5’-TGAAGGACAGGAATGGGAAC-3’ |  |
| GATA-3 | Forward | 5’-AGGGACATCCTGCGCGAACTGT-3’ | 166 |
|  | Reversed | 5‘-CATCTTCCGGTTTCGGGTCTGG-3’ |  |
| RORγt | Forward | 5’-GCGGCTTTCAGGCTTCATGGAG-3’ | 221 |
|  | Reversed | 5’-GGGCGCTGAGGAAGTGGGAAAA-3’ |  |
| Foxp3 | Forward | 5’-GAAGAATGCCATCCGCCACAAC-3’ | 114 |
|  | Reversed | 5’-ATTCATCTACGGTCCACACTGCTC-3’ |  |
| GAPDH | Forward | 5’-TGTGTCCGTCGTGGATCTGA-3’ | 150 |
|  | Reversed | 5’-TTGCTGTTGAAGTCGCAGGAG-3’ |  |

(6) PCR reaction: PCR reaction system was constructed : the corresponding components of PCR reaction were added to 200 μl PCR tube according to the following proportion, mixed and centrifuged quickly.

| 2×Taq PCR Master Mix | 12.5 µl |
| --- | --- |
| Forward Primer（10 µmol/L） | 1 µl |
| Reverse Primer（10 µmol/L） | 1 µl |
| cDNA template | 2 µl |
| dH_2_O | 8.5 µl |
| Total | 25 µl |

PCR reaction conditions:

94°C，3 min ×1 cycle

94°C，30 sec

60°C，15 sec

×35 cycles

72°C，15 sec

72°C，5 min ×1 cycle

4°C，for ever

After the PCR reaction, the PCR products were subjected to agarose gel electrophoresis. (7) Agarose gel electrophoresis: Preparation of gel: 10 ml 2 % agarose gel (containing 1 ‰ Goldview dye) was prepared according to the above method. After the agarose gel was slightly cooled, it was quickly poured into the gel groove of the sample comb, and the air bubbles between the comb teeth were checked. After the gel was cooled and formed, the sample comb was pulled out. Sample loading and electrophoresis: The prepared 2 % agarose gel was placed in a horizontal electrophoresis tank, poured into 1 × TBE buffer, and slightly passed the upper layer of the gel (about 1 ~ 2 mm). An 8 μl PCR reaction product or DNA Marker was added to the spotting hole. The electrophoresis tank power supply was connected, and the voltage was set to 100 V. The electrophoresis was stopped when the bromophenol blue shifted to 2 / 3 from the beginning of the gel tank, and the power supply was turned off. Gel imaging: The gel after electrophoresis was taken out and placed in an automatic gel imaging analysis system. The presence or absence of bands was observed under ultraviolet light irradiation, and the target band was judged according to the DNA Marker.

(8) Quantitative real-time PCR (qPCR) reaction: Experimental principle: qPCR is to add fluorescent substances to the PCR reaction system. During the amplification of the target product, the PCR process is monitored in real time by collecting and analyzing the fluorescence signal. In the qPCR program, two parameters are generally set: internal reference gene and reference factor. GAPDH or β-actin can be selected as the internal reference gene, but it is necessary to determine that the intervention factors in the experiment have no effect on the selected internal reference. The reference factor generally selects untreated samples. In this experiment, GAPDH was selected as the internal reference gene, and the target gene content of wild-type normal mouse lung tissue samples (control group) was used as the reference factor. The expression levels of the target genes in other groups ( experimental groups ) were calculated according to the 2- △ △ Ct method. qPCR reaction: Construction of qPCR reaction system: The corresponding components were added to the qPCR tube / plate according to the following ratio, mixed well and centrifuged quickly. At least 3 duplicate holes were made for each sample.

| SYBR^®^ Premix Ex Taq^TM^Ⅱ（2×） | 12.5 µl |
| --- | --- |
| PCR Forward Primer（10 µmol/L） | 1 µl |
| PCR Reverse Primer（10 µmol/L） | 1 µl |
| DNA templates | 2 µl |
| dH_2_O | 8.5 µl |
| Total | 25 µl |

The qPCR reaction conditions (two-step procedure) :

95°C，30 sec ×1 cycle

95°C，5 sec

×40 cycles（qPCR reaction）

60°C，20 sec

95°C，15 sec

×1 cycle Melt curve

60℃，60 sec（0.05°C/sec- 95°C）

95°C，15 sec

After the qPCR reaction was completed, the primer specificity was first judged according to the Melt curve, and then the qPCR results were analyzed by 2- △ △ Ct method. The specific steps were as follows : First, the Ct average value of the target gene and the internal reference gene of each sample was calculated, and then corrected with the internal reference gene GAPDH : △ Ct = the Ct average value of the target gene-the Ct average value of the GAPDH gene. Normalize the △ Ct of the target gene in wild-type normal mouse samples ( control group ) : △ △ Ct = △ Ct ( experimental group ) - △ Ct ( control group ). According to 2- △ △ Ct, the expression difference of the target gene between the experimental group and the control group was calculated.

**8.Enzyme-linked immunosorbent assay (ELISA)**

Coating: The coated antibody was diluted with 1 × coating solution according to the instructions, and then the coated antibody was added to a 96-well plate, 100 μl / well, and the plate was sealed overnight at 4 ° C. Washing plate: Take out the 96-well plate overnight at 4 ° C, and discard the coating solution containing the coating antibody. Add 300 μl of washing liquid to each hole, stand for 1 minute at room temperature, dry the liquid in the hole, and repeat the washing plate 5 times. Closure: 1 × detection buffer (containing 10 % FBS) was added to the 96-well plate, 200 μl / well, incubated at room temperature for 1 hour. After 1 hour, the liquid in the hole was dried, and the washing plate was repeated 5 times. Incubation of standard samples and samples to be tested: Standard samples and samples to be tested (stock solution or diluted samples) were added to 96-well plates in turn, 100μl / well, and blank control holes were set up, and incubated at room temperature for 2 hours. After 2 hours, the liquid in the hole was dried and the plate washing was repeated 5 times. Standard preparation: The standard was prepared according to the instructions, and 7 concentration gradients were set in a 1 / 2 decreasing manner. However, it should be noted that pre-experiment should be carried out in advance to determine that the concentration of the sample solution to be tested or the diluted sample concentration does not exceed the upper and lower limits of the standard. Incubation detection antibody: According to the instructions, the detection antibody was diluted with 1 × detection buffer, and then the detection antibody was added to a 96-well plate, 100μl / well, incubated at room temperature for 1 hour. After 1 hour, the liquid in the hole was dried, and the washing plate was repeated 5 times. Incubation of enzyme-labeled antibody: The avidin-linked HRP was diluted with 1 × detection buffer according to the instructions, and then HRP was added to a 96-well plate, 100 μl / well, and incubated at room temperature for 30 minutes. After 30 minutes, the liquid in the hole was dried and the plate washing was repeated 7 times. Color reaction: TMB was added to 96-well plates, 100 μl / well, incubated at room temperature for 10 ~ 15 minutes. Terminating reaction: 50 μl terminating solution (2 mol / L sulfuric acid) was added to the 96-well plate to terminate the reaction. Reading and analysis: After the termination of the reaction, the optical density (OD) value of each hole in the 96-well plate was detected by a microplate reader (the wavelength was set to 450 nm). The Reader Fit ELISA software was used for analysis, and the standard curve was drawn according to the OD value of the standard, and the sample concentration was calculated.

**9. Detection steps of ATP content in BALF**

Remove the ATP detection kit from the 4 °C refrigerator and balance it at room temperature in advance. Preparation of substrate solution (containing luciferase): 5 ml substrate buffer was accurately extracted and added to a tube of substrate by using a micropipette tip without ATP, and gently blew until the substrate was completely dissolved, and the solution was uniform. Preparation of ATP storage solution: A tube of ATP standard was taken, and the ATP standard was prepared into a storage solution with a concentration of 10 mmol / L with ultrapure water according to the dilution multiple marked on the tube wall. The ATP powder was completely dissolved by blowing several times, stored at-20 °C, and used within 1 month. Set the concentration gradient of ATP standard: set a total of 8 concentration gradients of 0 (blank control), 0.01,0.1,1,10,100,1000 and 10000 nmol / L (the concentration gradient range of ATP standard should be determined according to the preliminary experiment, generally in the range of 0 ~ 10 μmol / L). Ultrapure water was used to dilute 10 mmol / L ATP storage solution in proportion. A micropipette tip without ATP was used to absorb 100 μL BALF supernatant and add it to the 96-well plate to be tested. At the same time, 100 μl sterile PBS buffer for bronchoalveolar lavage was added to the 96-well plate standard hole. 50 μl of cell lysate (containing fluorescein) was sucked using a micropipette tip without ATP, and added to the sample hole and standard hole corresponding to the 96-well plate, and placed in the orbiting shaker for mixing (700 rpm × 5 min). A micropipette tip without ATP was used to absorb 10 μL of ATP standard with different concentrations, which was added to the above standard wells containing fluorescein, and placed in a fixed-track shaker for mixing (700 rpm × 5 min). A micropipette tip without ATP was used to draw 50 μl substrate solution (containing luciferase), which was added to the sample hole and standard hole containing fluorescein in the 96-well plate, and placed in the orbiting shaker for mixing (700 rpm × 5 min). The mixed reaction solution was kept away from light for 10 minutes, and the relative light unit (RLU) was detected by chemiluminescence instrument. The RLU standard curve was drawn according to different concentrations of ATP standard, and the ATP concentration of the sample to be tested was calculated.

10.Western blot (WB) detection

(1) Preparation of tissue protein samples: 50 ~ 100 mg mouse lung tissue blocks were placed in the spherical part of the homogenizer, and cut as much as possible with ophthalmic scissors. 500 μl protein lysate containing protease and phosphatase mixed inhibitor (50 ×) (RIPA) was added to the homogenizer, and homogenized intermittently on the ice for several times to fully crush the tissue. The tissue homogenate was transferred to a 1.5ml Eppendorf tube and allowed to stand on ice for 30 minutes. During this period, the tissue homogenate was shaken several times to fully lyse and release the protein. After standing for 30 minutes, centrifuged at low temperature (12000 g × 15 min, 4 ° C), and the supernatant was transferred to a new 1.5 ml Eppendorf tube, stored at − 80 ° C for later use. If the sample to be tested was bone marrow-derived dendritic cells (the third part of the test content), the semi-suspended dendritic cells were collected by centrifugation, and washed with PBS buffer once. The protein lysis buffer containing protease and phosphatase mixed inhibitors (50 ×) was added to each well of the 6-well plate according to the proportion of 150-200 μL lysis buffer, and then the cells were fully lysed by flicking the fingers. After centrifugation at low temperature (12000 g × 5 min, 4 ° C), the supernatant was transferred to a new 1.5 ml Eppendorf tube and stored at-80 ° C for later use.

(2) Determination of protein content by BCA method: According to the operation instructions of BCA protein detection kit, the main steps are as follows: According to the number of samples, an appropriate amount of BCA working solution was prepared according to the ratio of A solution : B solution = 50 : 1 ( volume ratio ), fully mixed, and used within 24 hours. 20 mg BSA protein standard was dissolved in 800 μl protein diluent to prepare 25 mg / ml standard protein solution. After fully dissolved, it was diluted with 0.9 % NaCl solution or PBS buffer to a protein working solution of 0.5 mg / ml. The standard substance was added to the standard hole of the 96-well plate at 0, 1, 2, 4, 8, 12, 16, and 20 μl, and the sample diluent was supplemented to 20 μl. The sample was pre-diluted 5 times with the sample diluent, and then 20 μl of the diluted sample solution was added to the sample hole of the 96-well plate. BCA working fluid (200 μl / well) was added to the standard and sample holes of the 96-well plate, and placed at 37 ° C for 30 minutes or at room temperature for 2 hours. Note : When the protein concentration was determined by BCA method, the absorbance value would deepen with time, and the color reaction would be accelerated due to the increase of temperature. Therefore, if the detected absorbance value is low, it can be incubated at a higher temperature or prolong the incubation time. The absorbance value of 570 nm wavelength was measured, and the total protein concentration in each sample was calculated according to the standard curve drawn by the standard substance.

(3) Sodium dodecyl sulfate-polyacrylamide gel electrophoresis (SDS-PAGE) electrophoresis: Clean the glass plate : gently scrub the two sides of the glass plate with a little washing powder, then rinse with running water, and finally rinse with distilled water, naturally dry. Glue filling: align the two glass plates and put them into the splint to clamp, and then vertically fixed on the shelf to prepare glue filling. Note: To ensure that the two glass plates are completely aligned during operation, so as to avoid leakage of glue, deionized water can be used in advance to detect whether there is leakage. According to the above method, 10 % separation adhesive was prepared. After adding TEMED, it was shaken immediately, and the adhesive could be filled. When the glue is poured, it is slowly poured along the inner wall of the glass plate to avoid bubbles in the glue. When the glue surface rises to the height of the middle line of the green belt (about 5.5 ml). Then, an appropriate amount of deionized water is added to the separation adhesive for liquid sealing, so that the separation adhesive can solidify faster. When there is an obvious refractive dividing line between water and glue, it indicates that the separation glue has solidified, and the upper deionized water can be poured out and the water can be dried by absorbent paper. The 4 % concentrated gel was prepared according to the above method, and immediately shaken up after adding TEMED, and the concentrated gel ( about 3 ml ) was slowly filled into the remaining space between the glass plates. Then the sample comb is vertically inserted into the unsolidified concentrated glue. After the concentrated gel was solidified, the two hands were kneaded on both sides of the sample comb, and gently pulled it out vertically. After washing with water, the glass plate was fixed in the electrophoresis tank. The small glass plate is facing inward, and the large glass plate is facing outward. After the protein content was measured, the volume of the solution containing 50 μg of total protein was calculated as the amount of protein loading. Before sample loading, the sample was taken out to 200 μl Eppendorf tube, and 5 × SDS loading buffer was added to the final concentration of 1 ×. The sample was boiled in boiling water for 5 ~ 10 minutes to make the protein completely denatured. Add a sufficient amount of electrophoresis buffer to the electrophoresis tank, at least through the inner small glass plate, and then start loading. When the sample was loaded, the sample was absorbed with a micro pipette, and then the pipette tip was inserted into the sample hole and slowly added. The total volume of the sample is generally not more than 15μl. Electrophoresis: The voltage of the concentrated gel was set to 55 V.When the bromophenol blue indicator ran to the boundary between the concentrated gel and the separation gel, the voltage was changed to 100 V.When the bromophenol blue ran to about 1 / 5 from the bottom of the separation gel, the electrophoresis was stopped. The total electrophoresis time was about 2 ~ 3 h. Film transfer: generally using wet transfer method. A. After electrophoresis, the glass plate containing glue was removed and rinsed with deionized water to remove the electrophoresis buffer on the surface. Pry open the glass plate, gently scrape off the concentrated gel, carefully peel off the separation gel, cut the target protein and the internal reference protein fragment according to the molecular weight of the protein Marker, and put it into the 4 ° C pre-cooled transfer membrane buffer. B. Polyvinylidene fluoride (PVDF) membranes of the corresponding sizes were cut, hydrated rapidly in methanol for several seconds, and then balanced in the transfer buffer. Cut the corresponding size of the filter paper, each piece of glue 6, the same balance into the membrane buffer. C. Open the transfer membrane clip to keep the black side (black as the negative electrode, white as the positive electrode) horizontal, and place it in the following order: sponge-three-layer filter paper-glue-PVDF membrane-three-layer filter paper-sponge. When placed, pay attention to avoid bubbles, so as not to affect the membrane, and the filter paper on both sides of the PVDF membrane can not contact with each other to avoid short circuit. D. Put the transfer film clip into the transfer film groove, the black side of the clip faces the black side of the groove, and the white side of the clip faces the red side of the groove. Because the heat will be generated during the electric transfer, the transfer tank needs to be placed in ice water. Generally, 270 mA cross-flow transfer is used for 1 hour. E. After electroporation, the PVDF membrane was immersed in 1 × ponceau red dye solution for 5 minutes ( shaken on a decolorization shaker ), and the excess dye solution was rinsed with running water to observe the protein on the membrane ( optional step ). If this step is done, the membrane should be washed three times with TBS-T buffer after observation). Immune response: A. Blocking: 5 % skim milk powder was prepared with TBS-T buffer as blocking solution. The PVDF membrane was immersed in TBS solution from bottom to top, then transferred to a plate containing blocking solution, and incubated for 2 hours on a decolorization shaker at room temperature. B. Incubation of primary antibody: Dilute the primary antibody to an appropriate concentration with a blocking solution (the optimal antibody concentration needs to be explored in advance, generally 1: 500 or 1: 1000). The PVDF membrane was taken out from the blocking solution, and the residual solution was removed with filter paper. The membrane protein was placed face down on the primary antibody surface and incubated at 4 ° C overnight. The next day, the membrane was washed with TBS-T buffer for 5 times, 10 minutes each time. C. Incubation of secondary antibody: The HRP-labeled secondary antibody was diluted with the blocking solution at 1: 5000, and the membrane protein was placed face down on the surface of the secondary antibody solution, and incubated on a decolorization shaker at room temperature for 1.5 hours. Then the membrane was washed with TBS-T buffer for 5 times, 10 minutes each time. Chemiluminescence, development: remove the cleaned PVDF membrane, drain the washing liquid on the filter paper, but do not make the membrane completely dry. Super ECL Plus ultrasensitive luminescent liquid was prepared and fully mixed according to the ratio of A liquid: B liquid = 1: 1 (volume ratio). The membrane protein was fully contacted with the hypersensitive luminescence solution, and immediately placed in an ECL chemiluminescence instrument. After exposure for 5 minutes, the band was observed and photographed. Results analysis: Image J image analysis software was used to analyze the gray value of the target protein and the internal reference protein bands.

**10. Preparation of mouse lung mononuclear cells**

The mice were routinely anesthetized with 1 % pentobarbital sodium. After the blood was taken from the eyeball, the chest cavity was opened, and the lung tissue was carefully separated and placed in 5 ml type I collagenase (working solution concentration of 1 mg / ml). The lung tissue was fully cut with an ophthalmic scissors and digested at 37 ° C for 1 hour. After digestion, it was fully blown away with a pasteurized pipette and filtered with a 200-mesh nylon mesh to obtain a single cell suspension of lung tissue. The 15 ml centrifuge tube was taken and 5 ml mouse lymphocyte separation solution was added. Then the single cell suspension of the above lung tissue was slowly added vertically to the lymphocyte separation liquid surface, and centrifuged at low temperature (2000 rpm × 25 min, 4 °C). After centrifugation, do not shake, carefully absorb the middle white membrane layer (single cell layer) with a Pasteur pipette, and wash twice with PBS buffer to obtain mouse lung mononuclear cells.

**11.The expression of CD39 in mouse lung dendritic cells was detected by flow cytometry.**

Mouse lung mononuclear cells were prepared according to the previous steps, and the cell concentration was adjusted to 1 × 107 cells / ml. 100 μl cell suspension was added to the flow tube, and Fc receptor blocking antibody ( anti-Mouse CD16 / CD32 monoclonal antibody, 1 μl / tube ) was added to avoid non-specific binding. PE-labeled CD11 c ( PE-CD11 c, 2.5 μl / tube ), APC / Cy7-labeled F4 / 80 ( APC / Cy7-F4 / 80, 5 μl / tube ), APC-labeled CD39 ( APC-CD39, 10 μl / tube ) or the corresponding isotype antibody were stained according to the instructions. The cells were stained in dark at 4 °C for 30 minutes, and then the cells were washed once with flow staining buffer and immediately detected. According to Bedoret D et al.[1], CD11c + F4 / 80-gate was set as the lung dendritic cell population ( CD11c was the surface marker of dendritic cells, F4 / 80 was the surface marker of macrophages, and CD11c and F4 / 80 were double-labeled to avoid macrophage interference ), and the mean fluorescence intensity ( MFI ) of CD39 molecules in this cell population was observed.

**12.Preparation of mouse bone marrow-derived dendritic cells (BMDC) and drug intervention**

The mice were sacrificed by cervical dislocation and immersed in 75 % alcohol for 15 minutes. The bilateral femurs and tibias of the mice were isolated under sterile conditions and placed in RPMI-1640 medium containing penicillin / streptomycin (100 ×). The culture medium was drawn with a 1 ml syringe, and the bone marrow cavity was punctured from one end of the diaphysis. The bone marrow was washed into another sterile culture dish and repeated 4-6 times. Then the mouse bone marrow cell suspension in the culture dish was collected and centrifuged at low temperature (1500 rpm × 7 min, 4 °C). After centrifugation, the supernatant was discarded, and 1 ml of red blood cell lysate was added to break the red. After fully blown, it was allowed to stand at room temperature for 7 minutes, and then 5 ml of RPMI-1640 medium containing 10 % FBS was added to dilute and terminate the reaction. Low temperature centrifugation (1500 rpm × 7 min, 4 ° C). The supernatant was discarded, and the cells were washed twice with PBS buffer. The cells were resuspended with 1 ml RPMI-1640 complete culture medium (RPMI-1640 + 10 % FBS + 1 % double antibody) and counted. RPMI-1640 complete medium was used to adjust the cell concentration of each group to 1 × 106 / ml, and the final volume was 10 ml. Finally, 10 μl rmIL-4 (1000 ×, final concentration 10 ng / ml) and rmGM-CSF (1000 ×, final concentration 10 ng / ml) were added to induce differentiation. The cells were cultured in a 37 °C, 5 % CO2 incubator for 7 days. Half of the medium was changed every other day. On the 7th day, the suspension cells enriched after gentle blowing were immature BMDC. In this experiment, WT normal mouse bone marrow-derived dendritic cells (WT-DC) and IL-27Rα- / -normal mouse bone marrow-derived dendritic cells (IL-27Rα- / -DC) were induced and cultured in vitro. On the 7th day of culture, rmIL-27 (500 ×, final concentration 20 ng / ml) and / or LPS (1000 ×, final concentration 10 ng / ml) were added for intervention. After 48 hours, the cells were collected for qPCR and western blot detection.

[1] Bedoret D, Wallemacq H, Marichal T, et al. Lung interstitial macrophages alter dendritic cell functions to prevent airway allergy in mice. J Clin Invest, 2009, 119(12): 3723-3738.

**Experiment Reagent**

1.Albumin from chicken egg white (Ovalbumin, OVA), Sigma-Aldrich Chemical Co., St. Louis, MO, USA

2.Immunoaluminum adjuvant (Imject Alum), Thermo Fisher Scientific Inc., Rockford, IL, USA

3.Pentobarbital Sodium, Beijing Solebold Biotechnology Co., Ltd., China

4.Genomic deoxyribonucleic acid (DNA) small amount extraction kit (centrifugal column), Shanghai Biyuntian Biotechnology Co., Ltd., China

5.Conventional PCR kit, Tiangen Biochemical Technology (Beijing) Co., Ltd., China

6.Agarose, Biowest S.A.S, Barcelona, Spain

7.Goldview Nucleic Acid Gel Stain (10000 ×), Beijing Solarbio Biotechnology Co., Ltd., China

8 DNA Marker (50 bp DNA ladder), Tiangen Biochemical Technology (Beijing) Co., Ltd., China

9. Acetyl-β-methylcholine chloride (Mch), Sigma-Aldrich Chemical Co., St. Louis, MO, USA

10. Bovine serum albumin (BSA), Sigma-Aldrich Chemical Co., St. Louis, MO, USA

11. Human anti-Mucin 5AC antibody, Abcam plc., Cambridge, MA, USA

12.Horseradish peroxidase (HRP) labeled Goat Anti-Human IgG (H + L) -HRP (secondary antibody), Bioworld Technology, Inc., St. Louis, MN, USA

13. DAB chromogenic kit, Wuhan Boster Bioengineering Co., Ltd., China

14.Total IgE and OVA-specific IgE ELISA Kit, eBioscience / Thermo Fisher Scientific Inc., San Diego, CA, USA

15. Cytokine ELISA Kits (IFN-γ, IL-4, IL-5, IL-13, IL-17A, IL-10), eBioscience / Thermo Fisher Scientific Inc., San Diego, CA, USA

16.ethylene diamine tetracetic acid (EDTA), Beijing Lingfei Technology Co., Ltd., China

17. Erythrocyte lysate, Beijing Solebold Biotechnology Co., Ltd., China

18. Wright-Giemsa Stain (Wright-Giemsa Stain) rapid detection kit, Nanjing Jiancheng Institute of Bioengineering, Jiangsu, China

19. Diethylpyrocarbonate (DEPC), Shanghai Biyuntian Biotechnology Co., Ltd., China

21. RNA reverse transcription kit (ReverTra Ace qPCR RT kit), Toyobo CO., LTD., Tokyo, Japan

22. Quantitative real-time PCR (qPCR), Toyobo CO., LTD., Tokyo, Japan

23.ATP detection kit (Luminescence ATP detection assay system), PerkinElmer, Inc., Boston, MA, USA

24. Antigen Affinity-purified Polyclonal Sheep IgG (Mouse CD39 / ENTPD1 Antibody), R & D Systems, Inc., Minneapolis, MN, USA

25.HRP-labeled Rabbit anti-Goat IgG (H + L) -HRP (secondary antibody), Bioworld Technology, Inc., St. Louis, MN, USA

26. Rat anti-human / mouse NLRP3 antibody (Human / Mouse NLRP3 / NALP3 Antibody, Monoclonal Rat IgG2A), R & D Systems, Inc., Minneapolis, MN, USA27. Rabbit anti-mouse ASC (apoptosis-associated speck-like protein containing a CARD, also known as TMS1 or PYCARD) antibody, Bioworld Technology, Inc., St. Louis, MN, USA

28. Rabbit anti-mouse Pro-caspase-1 / Caspase-1 antibody, Abcam, Inc., Cambridge, MA, USA

29. Rabbit anti-mouse IL-1β antibody (anti-IL-1beta antibody), Abcam, Inc., Cambridge, MA, USA

30. Rabbit anti-mouse IL-18 antibody, Abcam, Inc., Cambridge, MA, USA

31. HRP-labeled Goat anti-Rabbit IgG (H + L) -HRP (secondary antibody), Bioworld Technology, Inc., St. Louis, MN, USA

32. Radio immunoprecipitation assay buffer (RIPA buffer), Thermo Fisher Scientific Inc., Rockford, IL, USA

33.Protease phosphatase inhibitor mixture (50 ×), Shanghai Beyotime Biotechnology Co., Ltd., China

34. BCA protein assay kit, Wuhan Boster Bioengineering Co., Ltd., China

35. Skim milk powder, Beijing Solebold Biotechnology Co., Ltd., China

36. Super ECL plus detection reagent, Life Technology, Inc., Carlsbad, CA, USA

37. Cytokine ELISA kits ( IL-1β and IL-18 ), eBioscience / Thermo Fisher Scientific Inc., San Diego, CA, USA

38. Collagenase I, Sigma Chemical Co., St. Louis, MO, USA

39. Mouse Lymphocyte Separation Solution, Tianjin Haoyang Biological Products Technology Co., Ltd., China

Fc receptor blocking antibody (anti-Mouse CD16 / CD32 monoclonal antibody), Thermo Fisher Scientific Inc., San Diego, CA, USA

41.Phycoerythrin (PE) -labeled anti-mouse CD11c (Armenian Hamster IgG) and PE-labeled Armenian Hamster IgG isotype control, Thermo Fisher Scientific Inc., San Diego, CA, USA

42. APC / Cy7 anti-Mouse F4 / 80 (APC / Cy7 anti-Mouse F4 / 80, rat IgG2a, κ) and APC / Cy7 rat IgG2a, κ isotype control (APC / Cy7 Rat IgG2a, κ Isotype Control), BioLegend Inc., San Diego, CA, USA

Allophycocyanin (APC) labeled anti-mouse CD39 antibody (Mouse CD39 / ENTPD1 APC-conjugated antibody, monoclonal rat IgG1) and APC labeled rat IgG1 isotype control, R & D Systems, Inc., Minneapolis, MN, USA

Flow cytometry staining buffer, eBioscience / Thermo Fisher Scientific Inc., San Diego, CA, USA

45. Recombinant murine IL-4 (rmIL-4) and recombinant murine granulocyte-macrophage colony-stimulating factor (rmGM-CSF), Peprotech Inc., Rocky Hill, NJ, USA

46. Lipopolysaccharide (LPS), Sigma Chemical Co., St. Louis, MO, USA

47. Recombinant mouse IL-27 (rmIL27), R & D Systems, Inc., Minneapolis, MN, USA

48.5 ' -adenosine 5 ' -triphosphate (ATP), Sigma Chemical Co., St. Louis, MO, USA

49. Rabbit anti-mouse JAK1 antibody [ JAK1 (I1016) polyclonal antibody] and rabbit anti-mouse p-JAK1 antibody [ JAK1 (phospho-Y1022) polyclonal antibody], Bioworld Technology, Inc., St. Louis, MN, USA

50.rabbit anti-mouse JAK2 polyclonal antibody and rabbit anti-mouse p-JAK2 polyclonal antibody [ JAK2 (phospho-Y221) polyclonal antibody], Bioworld Technology, Inc., St. Louis, MN, USA

51. Rabbit anti-mouse JAK3 antibody [ JAK3 (I781) polyclonal antibody], Bioworld Technology, Inc., St. Louis, MN, USA

52. Rabbit anti-mouse p-JAK3 antibody [ phospho-JAK3 (Tyr980 / 981) monoclonal antibody], Cell Signaling Technology, Inc., Beverly, MA, USA

53. Rabbit anti-mouse TYK2 antibody [ TYK2 (V1048) polyclonal antibody], Bioworld Technology, Inc., St. Louis, MN, USA

54. Rabbit anti-mouse p-TYK2 antibody [ TYK2 (phosphor-Tyr1054) polyclonal antibody ], GeneTex, Inc., Irvine, CA, USA

55. Rabbit anti-mouse STAT1 polyclonal antibody and rabbit anti-mouse p-STAT1 polyclonal antibody [ Stat1 (phospho-Y701) polyclonal antibody], Bioworld Technology, Inc., St. Louis, MN, USA

56. Rabbit anti-mouse STAT2 antibody [ Stat2 (L684) polyclonal antibody] and rabbit anti-mouse p-STAT2 antibody [ Stat2 (phospho-Y690) polyclonal antibody], Bioworld Technology, Inc., St. Louis, MN, USA

57. Rabbit anti-mouse STAT3 polyclonal antibody and rabbit anti-mouse p-STAT3 polyclonal antibody [ Stat3 (phospho-S727) polyclonal antibody], Bioworld Technology, Inc., St. Louis, MN, USA

58. Rabbit anti-mouse STAT4 antibody [ Stat4 (E687) polyclonal antibody] and rabbit anti-mouse p-STAT4 antibody [ Stat4 (phospho-Y693) polyclonal antibody], Bioworld Technology, Inc., St. Louis, MN, USA

59. Rabbit anti-mouse STAT5 antibody [ Stat5a / b (A688) polyclonal antibody] and rabbit anti-mouse p-STAT5 antibody [ Stat5a / b (phospho-Y694) polyclonal antibody], Bioworld Technology, Inc., St. Louis, MN, USA

60. Rabbit anti-mouse STAT6 antibody [ Stat6 (G635) polyclonal antibody] and rabbit anti-mouse p-STAT6 antibody [ Stat6 (phospho-T645) polyclonal antibody], Bioworld Technology, Inc., St. Louis, MN, USA

61. RPMI-1640 medium, Gibco, Grand Island, New York, USA

62.Fetal bovine serum (FBS), HyClone, Logan, UT, USA

63. Penicillin / Streptomycin Mixture (100 ×), Beijing Solebold Biotechnology Co., Ltd., China
