# Supplementary material for: IL-27 Alleviates Airway Inflammation and Airway Hyperresponsiveness in Asthmatic Mice by Targeting the CD39/ATP Axis of Dendritic Cells
Source: Inflammation. 2023 Dec 20;47(2):807–21. doi: 10.1007/s10753-023-01945-9 (PMC11074049; doi:10.1007/s10753-023-01945-9)
Supplement: Supplementary file 2 — Supplementary file2 (DOCX 37988 KB) [file 10753_2023_1945_MOESM2_ESM.docx]

**Genotype identification of IL-27Rα knockout mice**

The results showed that the amplified band of WT mice was approximately 397 bp and the amplified band of IL-27Rα^- / -^mice was approximately 280 bp, which were consistent with the expectation. (Fig.1).


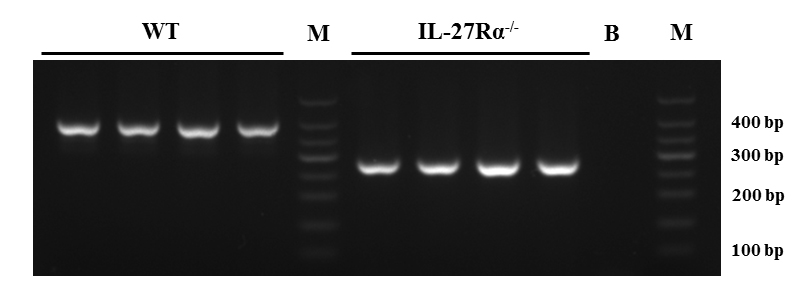


**Fig.1 Genotype identification of wild-type (WT) mice and IL-27Rα knockout (IL-27Rα- / -) mice. B : Blank ; m : DNA Marker ; WT : Wild Type**

| **a**  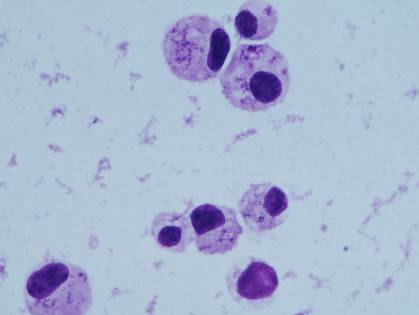 | **b**  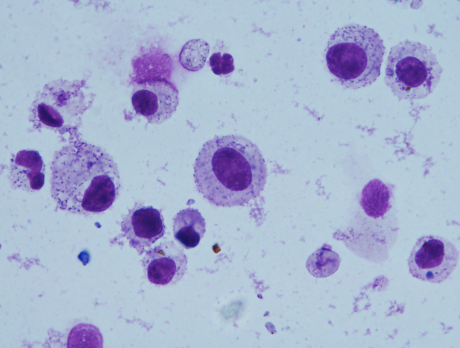 |
| --- | --- |
| **c**  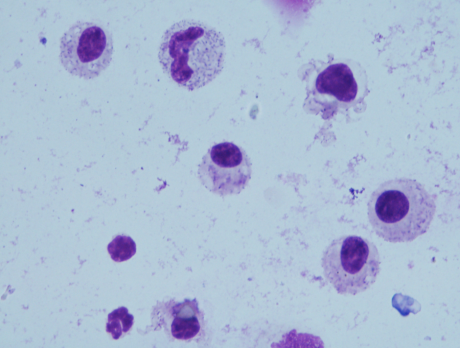 | **d**  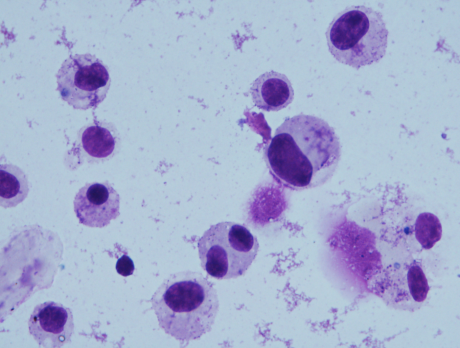 |

**Fig.2 Effects of IL-27Rα gene knockout on the total number and differential count of bronchoalveolar lavage fluid (BALF) cells in asthmatic mice. BALF cells of different groups of mice were stained by Wright-Giemsa and observed under a microscope (× 1000). a: WT normal mice, b: WT asthmatic mice, c: IL-27Rα- / -normal mice, d: IL-27Rα- / -asthmatic mice;**

| **A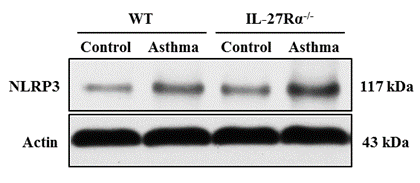** | | **B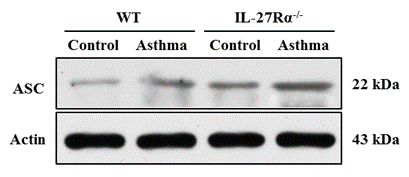** |
| --- | --- | --- |
| **C**  **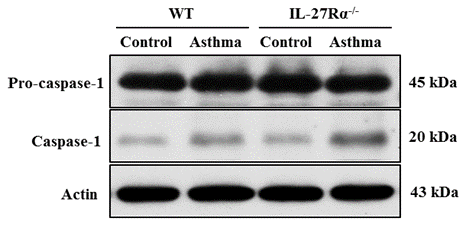** | | |
| **D**  **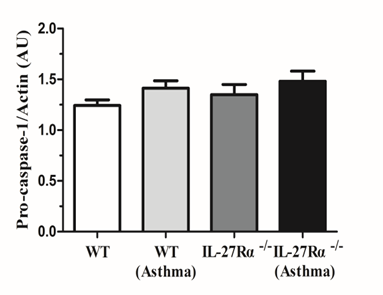** | **E**  **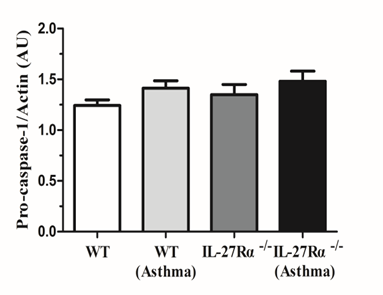** | |

**Fig.3 Effect of IL-27Rα gene knockout on the expression of NLRP3 inflammasome in lung tissue of asthmatic mice. A: Western blot was used to detect the expression of NLRP3 protein in lung tissue of mice in each group; B: Western blot was used to detect the expression of ASC protein in lung tissue of mice in each group. C,D and E: Western blot was used to detect the expression levels of Pro-caspase-1 and Caspase-1 protein in the lung tissue of mice in each group. * P < 0.05, * * P < 0.01, * * * P < 0.001.**

| **A**  **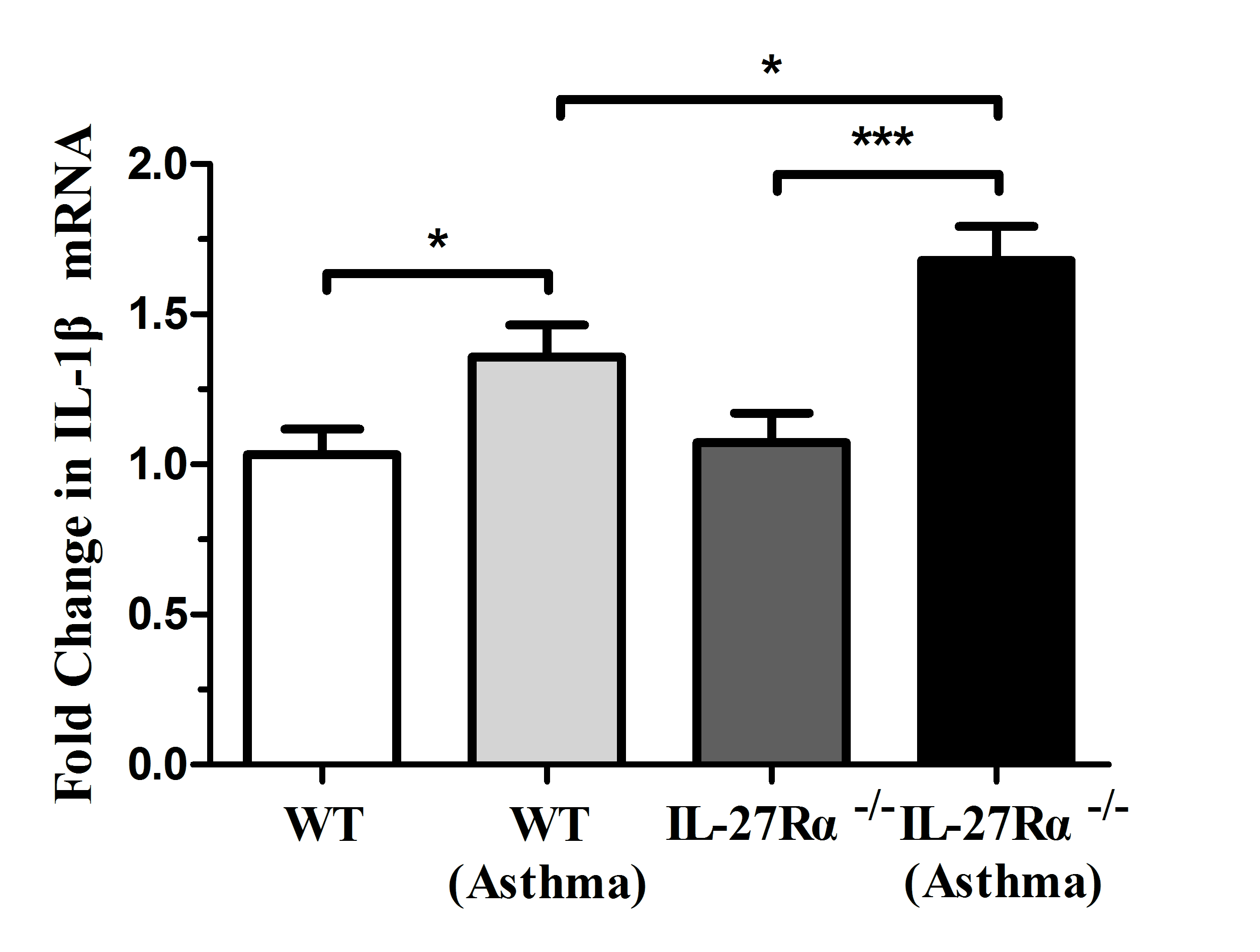** | **B**  **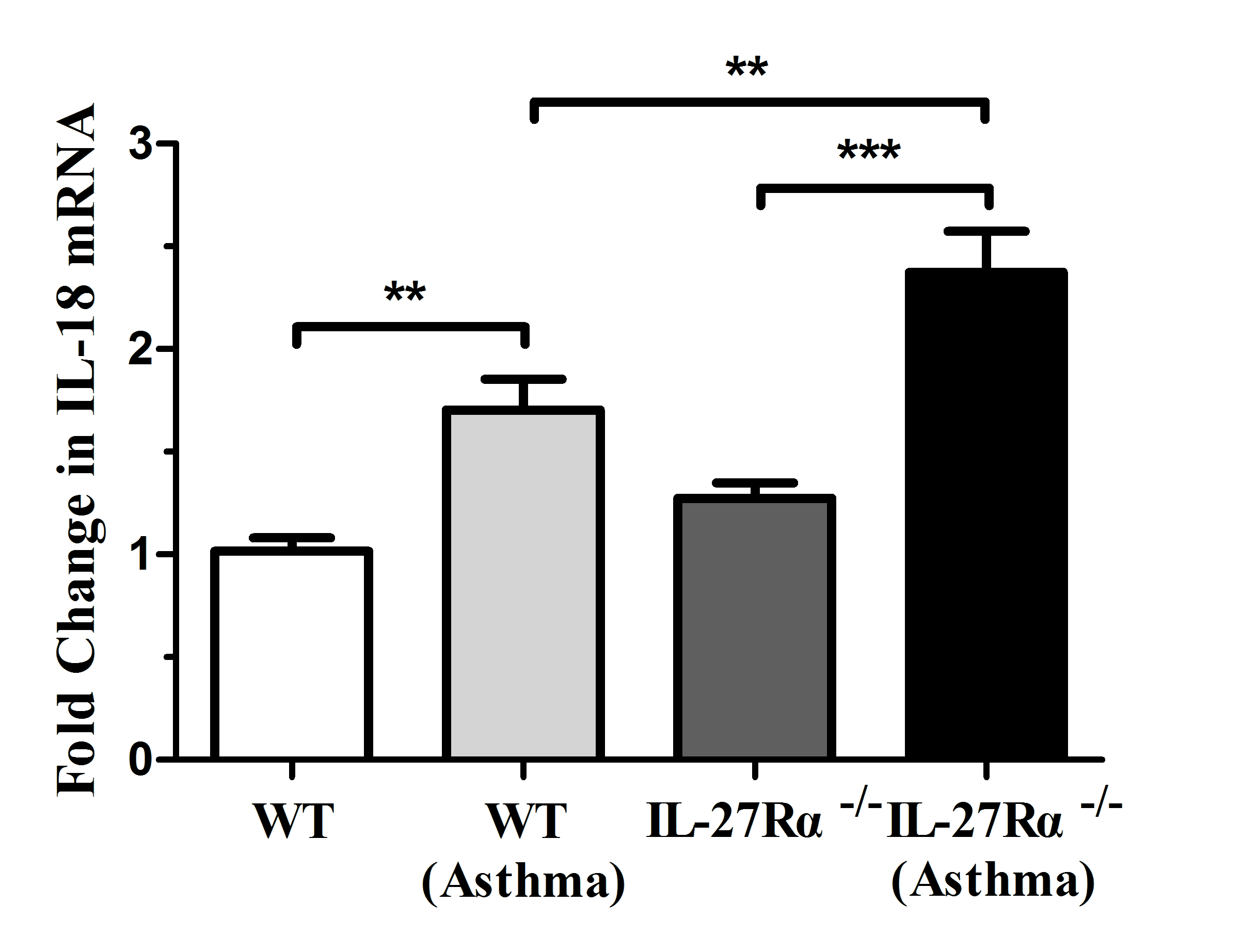** |
| --- | --- |
| **C**  **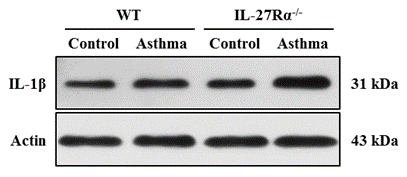** | **D**  **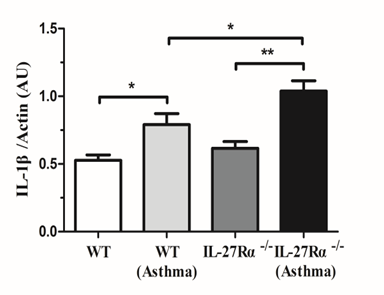** |
| **E**  **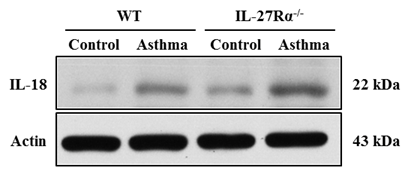** | **F**  **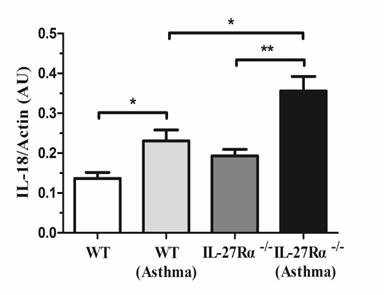** |
| **G**  **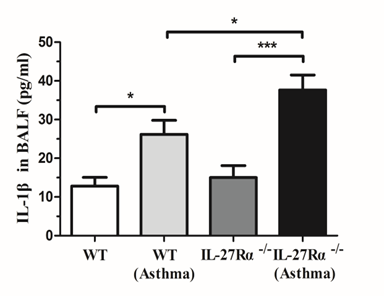** | **H**  **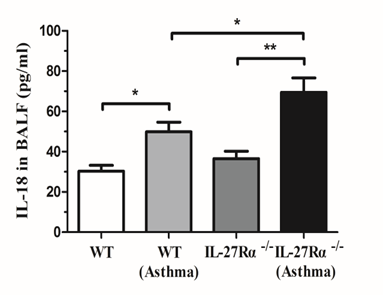** |

**Fig.4 Effects of IL-27Rα gene knockout on the expression of IL-1β and IL-18 in lung tissue and BALF of asthmatic mice. A and B: qPCR was used to detect the expression levels of IL-1β and IL-18 mRNA in lung tissues of mice in each group; C and D: Western blot was used to detect the expression of IL-1β protein in lung tissue of mice in each group; E and F: Western blot was used to detect the expression of IL-18 protein in lung tissue of mice in each group. G and H: The levels of IL-1β and IL-18 in BALF of mice in each group were detected by ELISA. * P < 0.05, * * P < 0.01, * * * P < 0.001.**

| **A**  **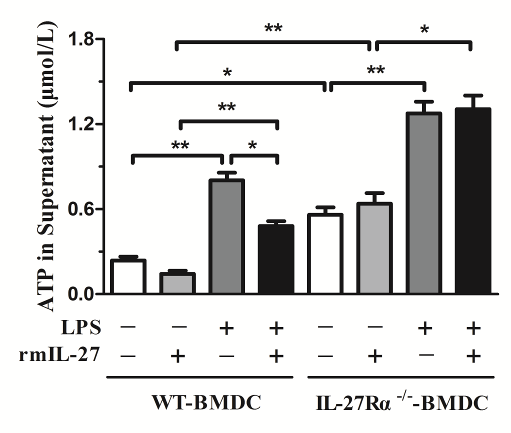** | **B**  **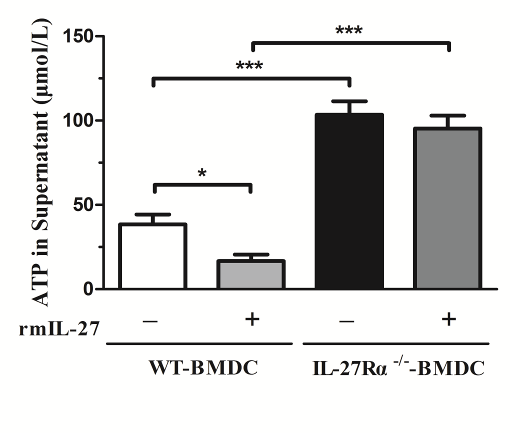** |
| --- | --- |

**Fig.5 Effect of IL-27Rα gene knockout on ATP hydrolysis function of BMDC in mice. A: ATP content in the supernatant of WT-BMDC and IL-27Rα- / -BMDC after 48 hours of LPS or (and) rmIL-27 intervention; B: ATP content in the supernatant of WT-BMDC and IL-27Rα- / -BMDC after hydrolysis of exogenous ATP ( 500μmol / L ) in the presence or absence of rmIL-27. * P < 0.05, * * P < 0.01, * * * P < 0.001.**

| **A**  **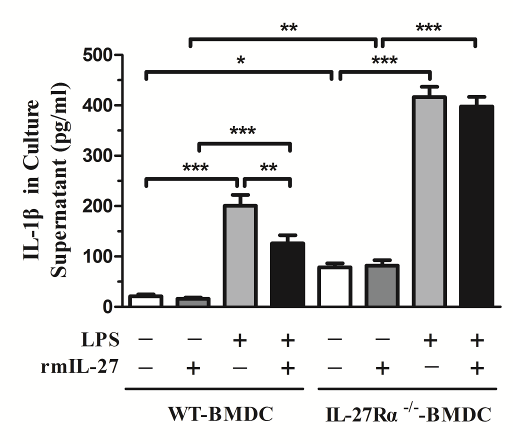** | **B**  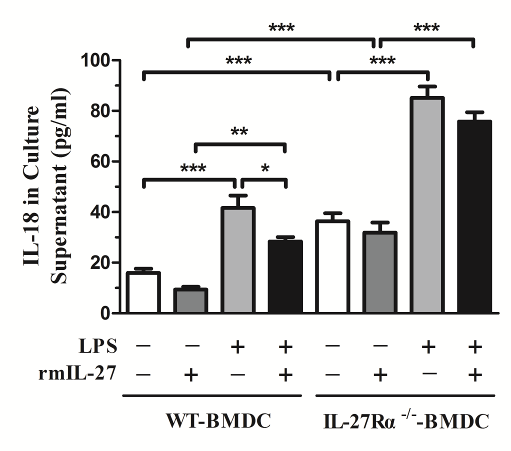 |
| --- | --- |

**Fig.6 The effect of IL-27Rα gene knockout on the content of IL-1β and IL-18 in BMDC culture supernatant. A: ELISA was used to detect the content of IL-1β in the supernatant of WT-BMDC and IL-27Rα- / -BMDC after 48 hours of rmIL-27 or ( and ) LPS intervention. B: ELISA was used to detect the content of IL-18 in the supernatant of WT-BMDC and IL-27Rα- / -BMDC after 48 hours of rmIL-27 or ( and ) LPS intervention. * P < 0.05, * * P < 0.01, * * * P < 0.001.**

| **A**  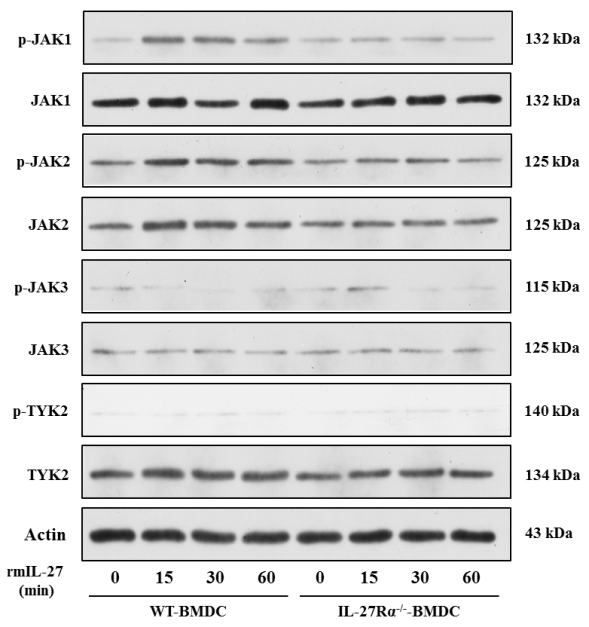 | |
| --- | --- |
| **B**  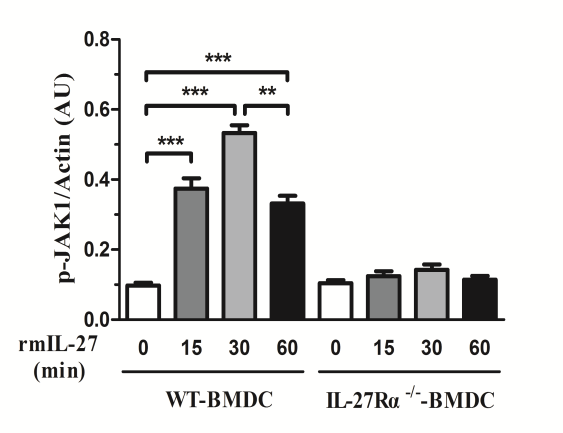 | **C**  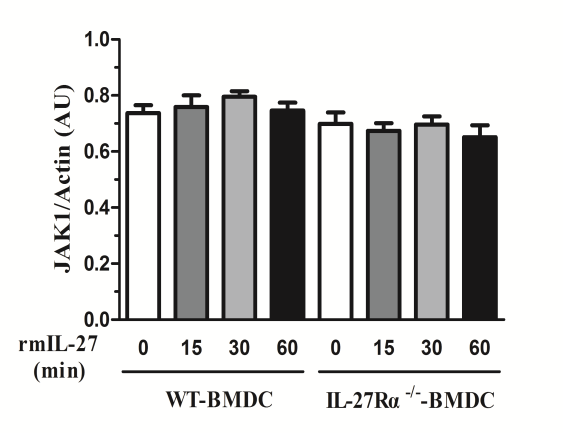 |
| **D**  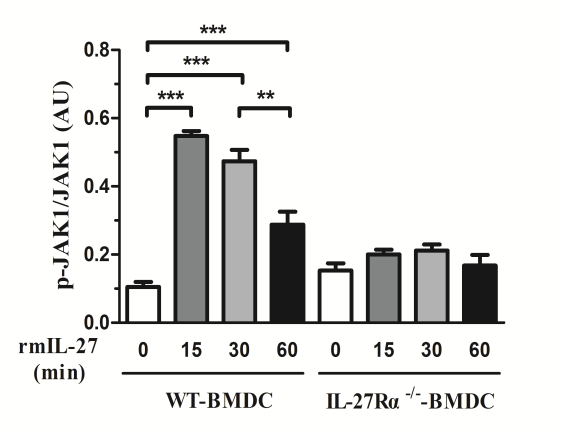 | **E**  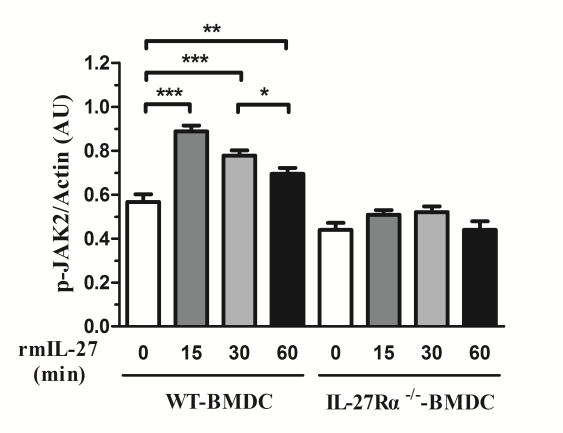 |
| **F**  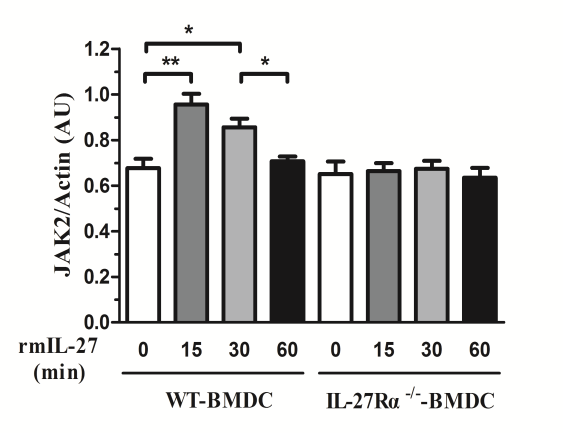 | **G**  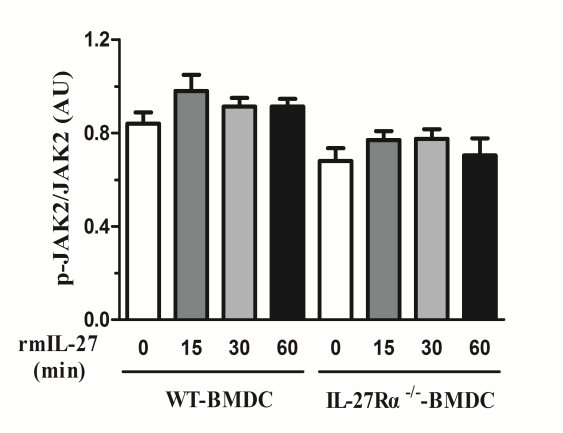 |
| **H**  **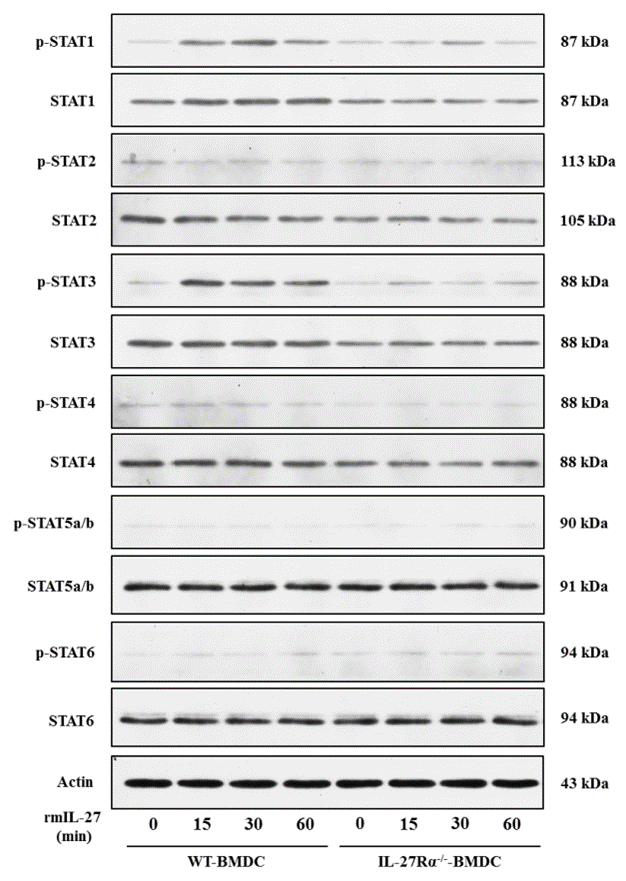** | |
| I  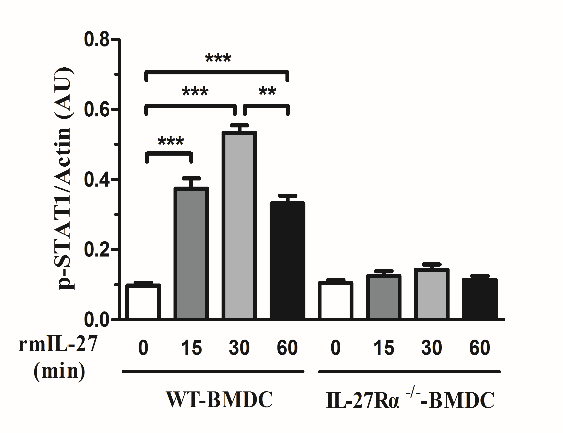 | **J**  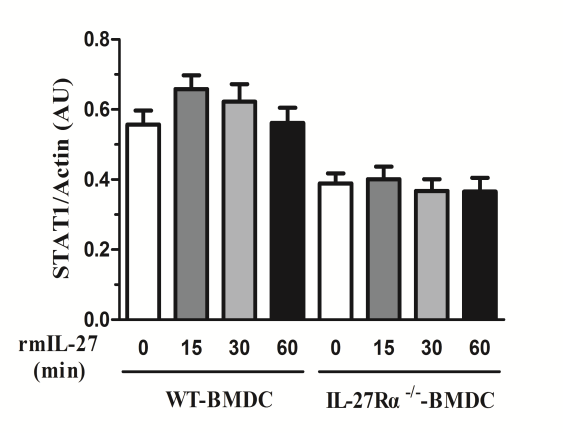 |
| K  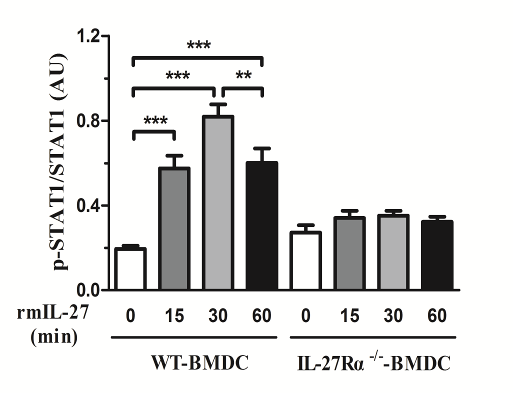 | L  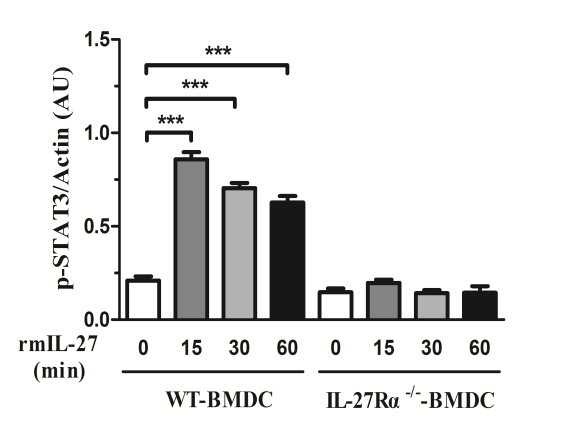 |
| **M**  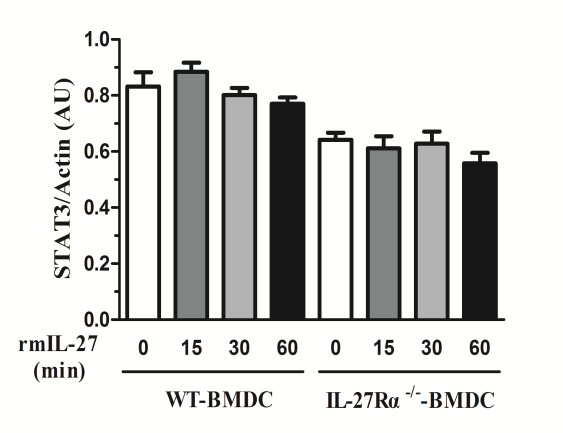 | N  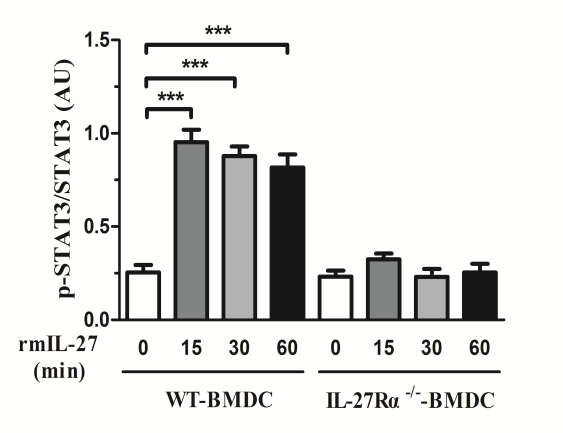 |

**Fig.7 Effect of IL-27Rα gene knockout on JAK / STAT signaling pathway in BMDC of mice. A: Western blot was used to detect the effect of IL-27Rα gene knockout on the expression and phosphorylation level of JAK family members (JAK1, JAK2, JAK3, and TYK2). B, C, and D: Western blot was used to detect the effect of IL-27Rα gene knockout on the expression of p-JAK1, JAK1, and p-JAK1 / JAK1 ratio. E, F, and G: Western blot was used to detect the effects of IL-27Rα gene knockout on the expression of p-JAK2, JAK2, and p-JAK2 / JAK2 ratio. H: Western blot was used to detect the effect of IL-27Rα gene knockout on the expression and phosphorylation of STAT family members ( STAT1, STAT2, STAT3, STAT4, STAT5, and STAT6 ). I, J, and K: Western blot was used to detect the effects of IL-27Rα gene knockout on the expression of p-STAT1, STAT1, and the ratio of p-STAT1 / STAT1. L, M, and N: Western blot was used to detect the effects of IL-27Rα gene knockout on the expression of p-STAT3, STAT3, and p-STAT3 / STAT3 ratio. * P < 0.05, * * P < 0.01, * * * P < 0.001.**
